# Mentalizing from self and friend perspectives: Relations between inter-brain neural similarity, affective similarity and social anxiety symptoms

**DOI:** 10.1162/IMAG.a.1403

**Published:** 2026-10-16

**Authors:** Alicia Vallorani, Kathryn A. McNaughton, Marisa N. Lytle, Michael N. Hallquist, Elizabeth Redcay, Koraly Pérez-Edgar

**Affiliations:** Department of Human Development and Quantitative Methodology, University of Maryland, College Park, MD, United States; Department of Psychology, The Pennsylvania State University, University Park, PA, United States; Department of Psychology, University of Maryland, College Park, MD, United States; Neuroscience and Cognitive Science Program, University of Maryland, College Park, MD, United States; Department of Psychology, North Carolina State University, Raleigh, NC, United States; Department of Psychology, University of North Carolina, Chapel Hill, NC, United States

**Keywords:** mentalizing, naturalistic neuroscience, inter-brain neural similarity, social anxiety

## Abstract

Social interactions are dynamic and complex, relying on tracking variation in your own and your partner’s affective and mental states. Being “in-sync” neurally and building a shared consensus can mark successful social interactions. In addition, social anxiety can impact social experiences. We used a novel, naturalistic paradigm to investigate relations between inter-brain neural similarity within mentalizing regions, affective similarity, and social anxiety symptoms. Undergraduate student friend pairs (N = 34, 85% White, 65% Women) engaged in a social interaction while videos captured from their individual perspectives were recorded. Participants watched clips of the social interaction from both their own perspective (their personal view of the social interaction) and their friend’s perspective (their friend’s personal view of the social interaction) while fMRI data were collected. They rated their affect after each clip. Inter-brain neural similarity was computed across three conditions: 1) Same-Stimuli: both participants viewed identical visual stimuli as in a traditional neural similarity paradigm, 2) Self-Perspective: both participants viewed the clip from their own perspective like the originally experienced social interaction, and 3) Friend-Perspective: both participants viewed the clip from their friend’s perspective, a novel perspective. Participants self-reported their social anxiety symptoms, and affective similarity captured affect rating concordance within dyads. In contrast to the Same-Stimuli condition, when participants viewed the clips like they originally experienced the social interaction (Self-Perspective), greater affective similarity was associated with greater inter-brain neural similarity. When participants viewed the clips from a novel perspective (Friend-Perspective), participants lower in social anxiety symptoms exhibited greater inter-brain neural similarity with greater affective similarity; whereas participants higher in social anxiety symptoms exhibited greater inter-brain neural similarity with less affective similarity. The results suggest stimuli from socially relevant perspectives, rather than identical stimuli, may reveal more nuanced brain-behavior dynamics, allowing for a better understanding of individual differences in socioemotional experience.

## 1 Introduction

Navigating social interactions is a core component of everyday life. Mentalizing, the ability to interpret the emotions and intentions of others (Frith & Frith, 2003), is one way people can track and respond to social interactions. Researchers have commonly studied mentalizing abilities from a third-person perspective, examining how people explicitly label differences in knowledge between themselves and others (Saxe, Moran, et al., 2006; Saxe, Schulz, et al., 2006). However, when people employ mentalizing skills during a natural social interaction, they do so spontaneously (Redcay & Schilbach, 2019) and in response to dynamic shifts within conversational and affective context, allowing interaction partners to understand each other in the moment. Thus, third-person tasks lack the nuance necessary to fully appreciate the role of the mentalizing network in supporting social interactions. It is imperative to use naturalistic methods to capture how characteristics of a social interaction, and social partner traits, impact mentalizing network dynamics within an interaction. The current study examines friend pairs as they view and assess a prior shared interaction, from their own perspective and their friend’s perspective, providing an opportunity to capture variation in inter-brain neural similarity tied to a shared social experience.

The mentalizing network is comprised of the mPFC, TPJ, posterior cingulate cortex/precuneus, hippocampus and STS (Jarcho & Guyer, 2018). The mPFC and TPJ are implicated in mental state detection (Frith & Frith, 2003), and also play an important role in self versus other distinctions. Evidence suggests the TPJ is associated more strongly with other-focused thought (Denny et al., 2012; Saxe, Moran, et al., 2006) while the mPFC often is associated with self-focused (Courtney & Meyer, 2020; Qin & Northoff, 2011), though the dmPFC also has been related to other-focused thought (Denny et al., 2012). Together, these processes allow partners to navigate social interaction dynamics. Previous work has used naturalistic methods to study the mentalizing network to understand socioemotional processing. For example, research suggests that the mPFC and TPJ show greater activation when viewing static images of social interactions versus non-social stimuli (Deuse et al., 2016). Additionally, watching movies of social interactions recruits the mentalizing network (Wagner et al., 2016; Wolf et al., 2010; Zaki et al., 2009). Work in children also suggests that the mentalizing network is differentially recruited when a person believes they are virtually interacting with a real person as opposed to reading statements about a third party (Alkire et al., 2018) or hearing a recorded voice (Rice & Redcay, 2016). Although these naturalistic methods allow for spontaneous mentalizing to occur, they still lack the dynamic nature of real-life social interactions. They also focus on an isolated individual.

Dual-brain approaches (Redcay & Schilbach, 2019; Wheatley et al., 2019) not only use naturalistic, interactive tasks to assess neural processing during social interactions, but also collect data from two brains involved in the same interaction. Data from dual-brain approaches can be collected simultaneously (Špiláková et al., 2019) or sequentially, as in work examining the transmission of affective states between partners (Anders et al., 2011). Stimuli used in sequential designs are derived from a shared task between two people, allowing for the examination of how two brains are responding to shared experiences. One metric that can be derived from dual-brain procedures is inter-brain neural similarity, a measure of the correspondence in neural processing of the same stimuli between two or more individuals. Inter-brain neural similarity in fMRI studies is commonly measured by having all participants view the same movie (an identical stimulus) and then correlating neural activity across participants to assess similarity in neural processing (Dmochowski et al., 2014; Finn et al., 2020; Popal et al., 2019). Participants who exhibit more inter-brain neural similarity may experience higher quality communication within study social interactions (Dziura et al., 2023) and be closer within a real-world social network (Parkinson et al., 2018). Additionally, people experiencing shared understanding (Nguyen et al., 2019; Yeshurun et al., 2017), memories (J. Chen et al., 2017) or emotional states (Nummenmaa et al., 2012) may experience higher levels of inter-brain neural similarity. Inter-brain neural similarity may mark people achieving common ground or finding a shared representation of their experience (Baek & Parkinson, 2022; Lahnakoski et al., 2014). Given the importance of the mentalizing network for recognizing intentions and emotions (Frith & Frith, 2003) and self- and other-focused thought (Denny et al., 2012; Saxe, Moran, et al., 2006), inter-brain neural similarity within the mentalizing network could be particularly sensitive to social interaction dynamics, such as shared affective states, or individual differences such as social anxiety.

Emotional expressions and affective experiences are integral components of social interactions that guide how interaction partners engage (Marinetti et al., 2011). Evidence suggests that partners who exhibit similar affective responses to shared experiences report greater social connection (Cheong et al., 2023). Additionally, adolescents who are closer within social networks exhibit more inter-brain neural similarity within the vmPFC, particularly for affectively salient stimuli (Feldman et al., 2024). Interestingly, both children at elevated social anxiety risk (Vallorani et al., 2022) and adults with social anxiety disorder (Pearlstein et al., 2019) are less likely to share positive affect, potentially reducing opportunities for social connection. Theory suggests that differences in mentalizing abilities during social interactions should be associated with the development of social anxiety (Jarcho & Guyer, 2018). However, research surrounding relations between mentalizing abilities, the mentalizing network, and social anxiety is muddied. Some evidence suggests social anxiety is related to reductions in mentalizing skills (Ballespí et al., 2018), potentially due to excessive self-focused thought (Boehme et al., 2015), while other work suggests social anxiety might be related to over-mentalizing, or thinking *too* much about what others are thinking (Ballespí et al., 2019; Washburn et al., 2016). However, this previous work has not assessed mentalizing abilities or the mentalizing network during live social interactions, limiting the understanding of how mentalizing abilities and the mentalizing network functions in the very context socially anxious individuals are thought to experience difficulties with mentalizing skills (Jarcho & Guyer, 2018).

The goal of the current study was to use a naturalistic neuroscience paradigm to better understand mentalizing network inter-brain neural similarity in the context of a real-life social interaction as moderated by social interaction dynamics (affective similarity) and individual differences (social anxiety symptoms). We had friend pairs come to our lab and engage in a naturalistic social interaction while their view of the social interaction was recorded using mobile eye-tracking glasses (Fig. 1A – Social Interaction). After the social interaction, we extracted moments from each participant’s view and had participants “re-live” those moments in the scanner from both their own and their friend’s view (Fig. 1A – Scanner Task).

**Fig. 1.**
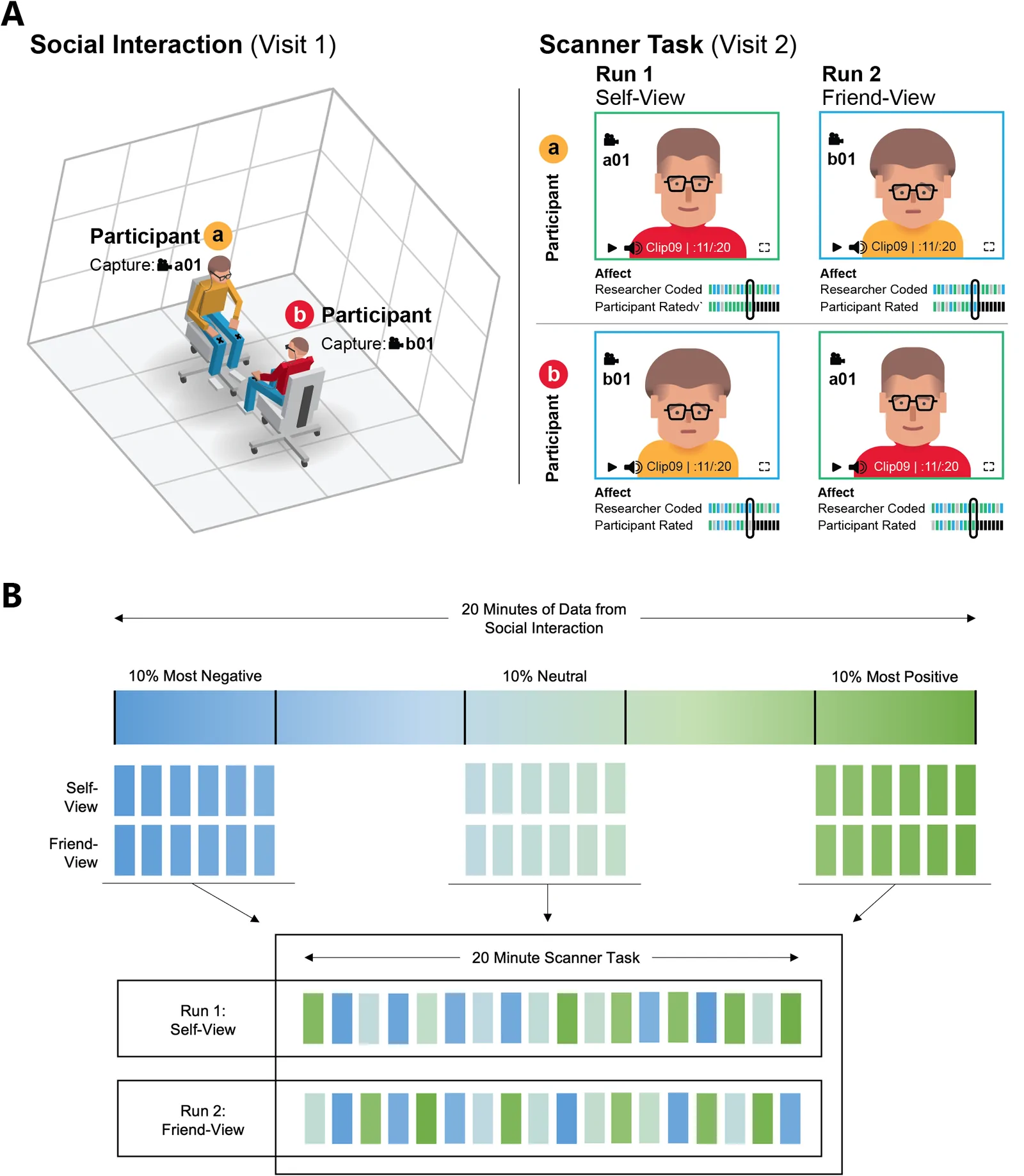
Visualization of procedures and stimuli creation. (A – Social Interaction) Participants engaged in a social interaction while their perspectives were recorded via mobile eye-trackers. (A – Scanner Task) Participants completed the BFFs Task while imaging data were collected. Participants watched clips from the social interaction that were previously coded as positive, negative, or neutral (B) from their own (Self-Perspective Run) and then from their friend’s (Friend-Perspective Run) perspective. Dyads watched the same clips in each run, just whose perspective it was (self vs. friend) changed. After each clip, participants rated how positive and how negative they felt.

Our method provided a unique opportunity to explore inter-brain neural similarity dynamics within the mentalizing network in relation to a naturalistic social interaction. First, in most inter-brain neural similarity paradigms, everyone watches the same movie and inferences about inter-brain neural similarity are made based on how neural activity correlates across participants who all took a singular, third-person perspective. Here, we assess how neural activity correlates within dyads across three different perspectives (Fig. 2). Participants took the same view (Same-Stimuli condition) when they viewed identical clips, matching visual input, even if the clip was from their own or their friend’s view. Participants took the self-view (Self-Perspective condition) when both participants viewed the clips from their own perspective (i.e., the same way they originally had experienced the social interaction). This view recreates the participant’s prior memory, but the similarity metric is based on non-identical stimuli. Participants took the friend view (Friend-Perspective condition) when both participants viewed the clips from their friend’s perspective (i.e., viewing themselves in the interaction). In this condition, along with non-identical stimuli, we now alter the original sensory input from the interaction.

**Fig. 2.**
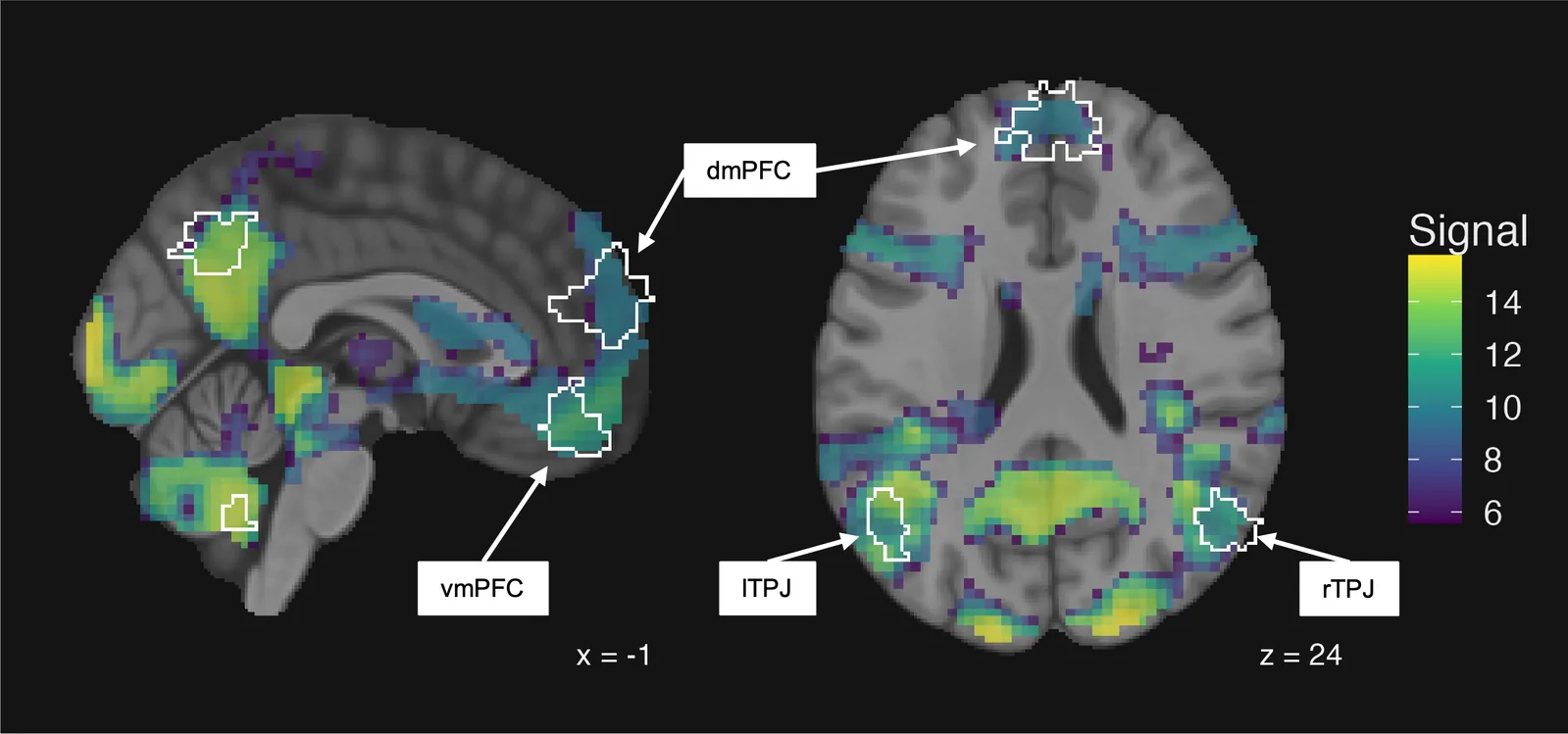
Whole-brain signal corrected at the *p* = .05 level. White outlines represent retained clusters. Labeled clusters represent clusters included in the multilevel models.

The design allowed us to compare inter-brain neural similarity when visual stimuli were identical (Same-Stimuli) against inter-brain neural similarity when visual stimuli were not identical and instead matched in perspective (Self-Perspective and Friend-Perspective). The Self-Perspective allowed us to examine inter-brain neural similarity in the context in which the social interaction was experienced. Conversely, the Friend-Perspective condition would be a novel experience to participants as people generally do not get to view the world through an interaction partner’s eyes. Given models suggesting socially anxious individuals may have more rigid attentional filters (Pérez-Edgar, 2018), we thought viewing the interaction through a friend’s view may elucidate potential individual differences in socioemotional experience. We also assessed if affective similarity (how similarly participants in each dyad rated their affect for a given clip) or social anxiety symptoms moderated inter-brain neural similarity between friends. Finally, because we had participants watch the social interaction both from their own and their friend’s perspective, we were able to probe potential self versus other distinctions within the mentalizing network.

By nature of the novel approach, our investigation is necessarily exploratory. However, based on previous research, we did anticipate that affective similarity would positively relate to inter-brain neural similarity in mentalizing regions (Nummenmaa et al., 2012). Additionally, we anticipated that higher levels of social anxiety symptoms might alter this relation due to either elevated levels of self-focused thought (Boehme et al., 2015) or over-mentalizing (Ballespí et al., 2019; Washburn et al., 2016). We also expected that if regional differences emerged that the mPFC would relate to self-representations (effects within the Self-Perspective condition) and the TPJ would relate to other representations (effects within the Friend-Perspective condition).

## 2 Method

### 2.1 Study overview

Young adult participants attended a laboratory visit with a platonic, same gender identity friend. They engaged in guided conversation for 20 minutes while mobile eye-tracking data were collected. Participants also completed a series of questionnaires after the conversation. Participants returned to the laboratory for a later scanning session. During the scanning session, they viewed clips from their previous social interaction from their own and their friend’s views (as collected by our mobile eye-tracking system). Participants rated how positively and how negatively they felt after each clip. After the scan, participants again completed a series of questionnaires. Informed consent was obtained from each participant. All procedures were approved by The Pennsylvania State University Institutional Review Board. Data are available on Databrary (Vallorani et al., 2023). Analytic code is available on GitHub (Vallorani, 2024).

### 2.2 Participants

Participants were screened for inclusion criteria prior to recruitment (Supplementary Section S1). Individuals who met inclusion criteria were welcome to participate with a platonic, same gender identity friend who met all the same criteria. 66 young adults (33 dyads) participated in the study. 34 young adults (17 dyads) provided data for the current analyses (see Supplementary Section S2 for information on excluded participants). Table 1 provides demographic information for the 34 participants included in the current analyses.

**Table 1.** Included participants’ demographics.

	N	*M (SD)*
Sex assigned at birth		
Female	22	
Male	12	
Gender identity		
Woman	22	
Man	12	
Sexual identity		
Heterosexual	28	
Bisexual	3	
Gay/lesbian	2	
Another identity	1	
Race		
White	29	
Asian	3	
Black	2	
Ethnicity		
Non-Hispanic	26	
Hispanic/Latine	4	
Another ethnicity	2	
Unknown	2	
Handedness		
Right	30	
Left	4	
Age at visit 1	34	19.79 (1.40)
Age at visit 2	34	19.84 (1.40)
Weeks between visits	34	2.90 (0.99)
Romantic attraction to friend	33	1.03 (0.17)
Sexual Attraction to friend	33	1.03 (0.17)

*Note*. *M* = mean; *SD* = standard deviation. Romantic and sexual attraction were rated on a 1–5 scale (1 = not at all attracted to 5 = very much attracted).

### 2.3 Procedures

#### 2.3.1 Visit 1

Participants engaged in a 20-minute guided conversation with their friend (Fig. 1A – Social Interaction). Participants were prompted to alternate who was leading the conversation every 5 minutes. For the first 10 minutes, participants were prompted to discuss something they were currently excited about in their lives. For the second 10 minutes, participants were prompted to discuss something they were currently worried about in their lives. Participants were recorded via a third-person wall mounted camera which faced both participants who were seated in chairs which faced the camera and were rotated approximately 30 degrees toward the other participant.

Individual views were recorded through head-mounted mobile eye-trackers. Prior to beginning the conversation, participants were informed by the researcher that “although you are being recorded, we will not have sound on in the back room while you are engaged in conversation and will not know what you are talking about while you are here today.” This procedure was undertaken to give participants privacy during the conversation. Researchers had a view into the room in case participants needed to signal for them for any reason. After the social interaction, participants completed questionnaires on their impressions of the social interaction, their friendship and their social anxiety symptoms.

#### 2.3.2 Visit 2

Participants attended a second visit in which they viewed the video clips from their previous social interaction (Fig. 1B – Scanner Task) while fMRI data were collected. The first run of the scan was always from the participant’s view, and the second run of the scan was always from their friend’s view. Between each video clip, participants rated how positive and how negative they felt. Each run of the task took approximately 10 minutes. After the scan was complete, participants again reported their impressions of the social interaction and reported their level of discomfort watching themselves during the scan.

### 2.4 Scanner task stimuli

During the social interaction, participant perspective videos were recorded using Pupil Invisible Glasses, a head-mounted mobile eye-tracker (PUPIL; Kassner et al., 2014; details about the system are included in Supplementary Section S3). Third-person videos of the social interaction were synced with the individual perspective videos in Pupil Player Version 3.5.7. Post syncing, the individual perspective videos and the third-person videos were exported separately. The third-person videos, with individual perspective videos embedded, were coded at a broad level for negative and positive affect (see Supplementary Section S3 for details). 18 clips were selected for each dyad to represent moments of negative, neutral, and positive affect for that dyad (Fig. 1B). These clips were exported from each participant’s perspective to use in the scanner task. Clips were then separated into two runs. The first run included clips taken from the participants’ own perspective (Self-Perspective Run), and the second run included clips taken from their friends’ perspective (Friend-Perspective Run). Clip order within the task was pseudo-randomized so that no two clips coded as the same affect occurred sequentially. Within dyad, presentation order was consistent such that participant a’s Self-Perspective Run order matched participant b’s Friend-Perspective Run order and vice versa.

### 2.5 fMRI data acquisition and processing

#### 2.5.1 fMRI acquisition

Researchers scanned participants using a Siemens 3T Magnetom Prisma Fit Scanner. First, an anatomical image was acquired using a sagittal MPRAGE T1-weighted sequence, voxel size = 1 mm^3^, TR = 2300 ms, TE = 2.96 ms, and flip angle = 9°. Next, participants completed the scanner task across two runs while BOLD functional images were acquired using a multislice sequence, voxel size = 2.7 mm^3^, TR = 635 ms, TE = 30 ms, flip angle = 49°, and multiband acceleration factor = 6. Participants always completed the self-run first followed by the friend run. Finally, we acquired gradient echo fieldmap images (TEs = 49.00 and 58.00 ms), allowing us to account for inhomogeneity of the magnetic field across the brain for each participant.

#### 2.5.2 fMRI preprocessing in fMRIPrep

Results included in this manuscript come from preprocessing performed using fMRIPrep 23.0.2 (Esteban et al., 2019; Esteban, Oscar et al., 2022), which is based on Nipype 1.8.6 (Gorgolewski et al., 2011). Boilerplate text automatically generated by fMRIPrep with the express intention that users should copy and paste this text into their manuscripts unchanged is included in Supplementary Section S4. It is released under the CC0 license. Below is an abbreviated version.

##### 2.5.2.1 Preprocessing of B0 inhomogeneity mappings

A B0-nonuniformity map (or fieldmap) was estimated based on two (or more) echo-planar imaging (EPI) references with topup (Andersson et al., 2003; FSL 6.0.5.1:57b01774).

##### 2.5.2.2 Anatomical data preprocessing

The T1-weighted (T1w) image was corrected for intensity non-uniformity and skull-stripped. Brain tissue was segmented, and brain surfaces were reconstructed and spatially normalized.

##### 2.5.2.3 Functional data preprocessing

A reference volume and its skull-stripped version were generated by aligning and averaging 1 single-band references (SBRefs). Head-motion parameters were estimated. The estimated fieldmap was then aligned with rigid-registration to the target EPI reference run. The field coefficients were mapped on to the reference EPI using the transform. BOLD runs were slice-time corrected to 0.274 s (0.5 of slice acquisition range 0 s-0.547 s) and then co-registered to the T1w reference with six degrees of freedom. Several confounding time-series were calculated based on the preprocessed BOLD: framewise displacement (FD), DVARS, and three region-wise global signals (CSF, the WM, and the whole-brain masks). Head-motion estimates calculated were calculated and frames that exceeded a threshold of 0.5 mm FD or 1.5 standardized DVARS were annotated as motion outliers. The BOLD time-series were resampled into standard space, generating a preprocessed BOLD run in MNI152NLin2009cAsym space.

##### 2.5.2.4 Post fMRIPrep preprocessing

fMRIPrep outputs were visually inspected to confirm segmentation, normalization, field map application, and alignment between structural and functional scans. Participants included in the current analyses remained adequately still throughout the scan (max movement *M_self_* = 1.13 mm, *SD_self_* = 0.88 mm; mean movement *M_self_* = 0.16 mm, *SD_self_* = 0.05 mm; max movement *M_friend_* = 1.21 mm, *SD_friend_* = 0.86 mm; mean movement *M_friend_* = 0.16 mm, *SD_friend_* = 0.05 mm). Post visual inspection, data were smoothed using a 6 mm full-width at half-maximum Gaussian filter and ICA AROMA was applied to handle movement artifacts. Data were then filtered by applying a 0.0083 Hz temporal high-pass filter. Finally, data were intensity normalized setting the global median to 1000.

##### 2.5.2.5 Whole-brain analyses and deconvolution

A voxelwise GLM analysis was conducted using FSL and fmri.factory (Hallquist, 2022) for levels 1 (individual), 2 (run) and 3 (group). Overall signal in the level 3 group brain map was inspected to confirm signal matched general expectations (ex: confirmation of signal in visual cortex). Whole-brain analyses were FWE corrected (*p* < .05) via pTFCE (Spisák et al., 2019) and clusterized via AFNI. Clusters were subsetted against the Neurosynth mentalizing atlas (Yarkoni et al., 2011; minimum overlap = 0.60; Fig. 2). The mentalizing atlas was obtained by searching “mentalizing” under “term-based meta-analyses.” We extracted the “association test” map and kept Neurosynth’s default thresholding (FDR criterion = 0.1). We estimated real-time voxelwise activity within retained clusters via deconvolution (Bush & Cisler, 2013) and time-locked to the start of each clip, creating a time series of activity for each region for each trial. For the current analyses, we focused on the time series from the dmPFC, vmPFC, rTPJ, and lTPJ (Supplementary Table S1). We focused on these regions given previous work highlighting the role of the mPFC (Courtney & Meyer, 2020; Qin & Northoff, 2011) in self-representations and the TPJ (Denny et al., 2012; Saxe, Moran, et al., 2006) in other-representations and our interest in examining if there were any potential self vs. other effects by region.

### 2.6 Measures

#### 2.6.1 Inter-brain neural similarity

Inter-brain neural similarity is typically assessed from imaging data obtained while participants watch the same movie. Here, we had the unique opportunity to compare inter-brain neural similarity when visual stimuli were identical against inter-brain neural similarity when the social context, but not the visual stimuli, was consistent. Thus, we had 3 perspective conditions (Fig. 3): Same-Stimuli (identical visual stimuli), Self-Perspective (visual stimuli from each participant’s own perspective), and Friend-Perspective (visual stimuli from each participant’s friend’s perspective). Time series extracted from the deconvolution were aligned across these conditions, and Pearson’s correlations were computed to assess inter-brain neural similarity for each clip. Higher values represent greater inter-brain neural similarity. Supplementary Figures S1–S4 provide an example of aligned deconvolved signal for one dyad across perspective conditions.

**Fig. 3.**
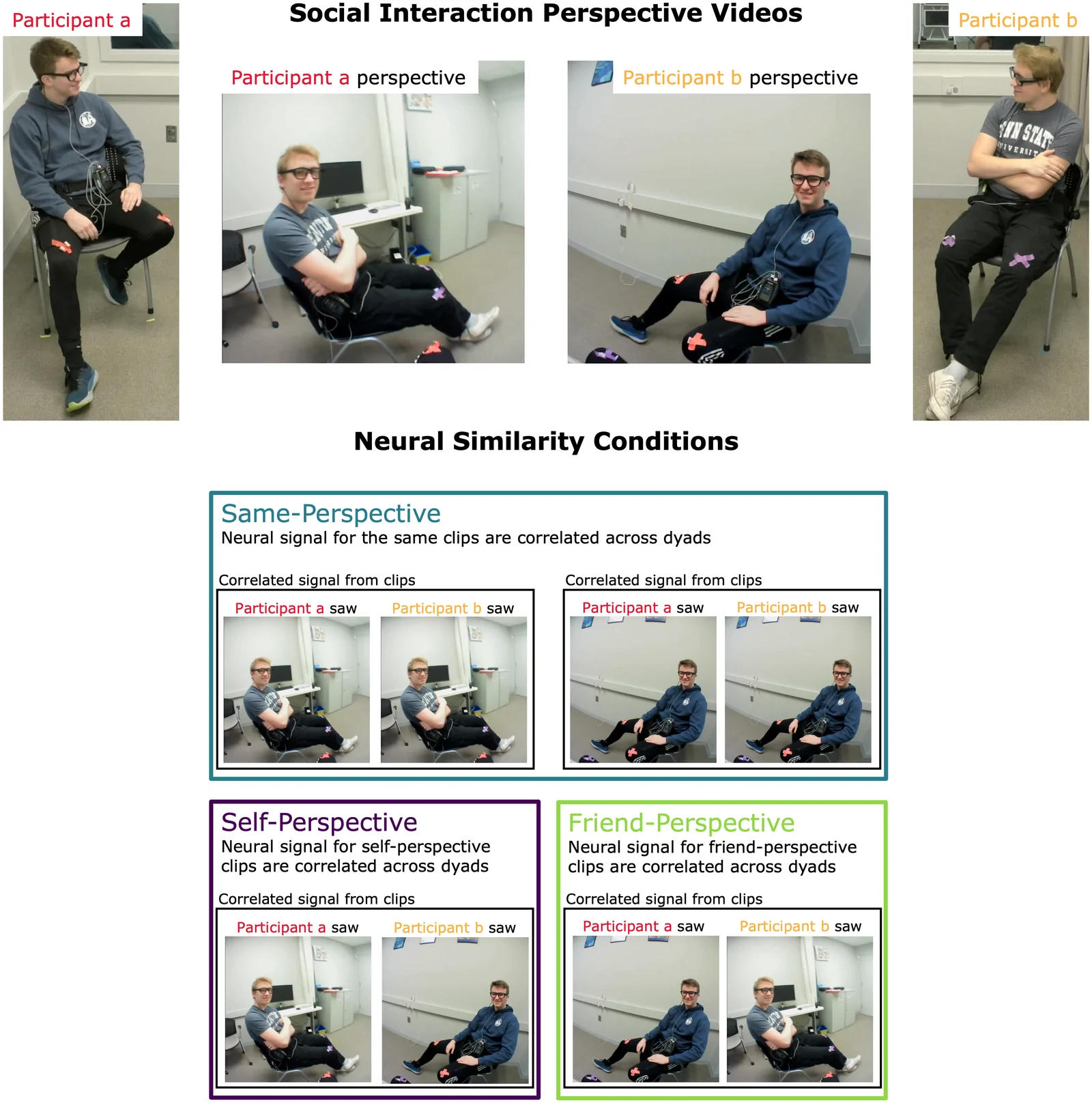
Creation of neural similarity conditions. Participant perspective videos taken during the social interaction were used to create the scanner task which comprised the Self-Perspective Run (clips from a participant’s own perspective) and the Friend-Perspective Run (clips from the participant’s friend’s perspective). Neural signal was correlated across and within these runs to create three neural similarity conditions. In the Same-Perspective condition, neural signals from the same clips were correlated across dyads. In the Self-Perspective condition, neural signals from clips from each participant’s self-perspective run were correlated across dyads. In the Friend-Perspective condition, neural signals from clips from each participant’s friend-perspective run were correlated across dyads.

#### 2.6.2 Affective similarity

After viewing each clip during the scanner task, participants responded to the statement “I feel positive” followed by the statement “I feel negative” on a scale from 0 – not at all to 4 – very. Responses were recorded so that negative remained 0–4 and positive became 5 – not at all to 8 very. A difference score was computed for each participant by subtracting how negative they felt for a given clip from how positive they felt for a given clip, meaning higher scores represented feeling more positive than negative affect for a given clip. Affective similarity was computed for each dyad for each clip by multiplying the absolute value of the difference between each friend’s rating by -1. Thus, higher scores represent greater affective similarity. Affective similarity was then decomposed into trait and state components for the purposes of modeling the multilevel data. Trait affective similarity was computed as each dyad’s mean affective similarity (*range* = -1.87 to -0.68), and state affective similarity was computed by subtracting trait affective similarity from the affective similarity score for each clip *(range* = -4.03 to 1.87)*.*

##### 2.6.2.1 Social anxiety symptoms

Participant social anxiety was assessed via the 24-item Lebowitz Social Anxiety Scale (Liebowitz, 1987). For each item, participants indicated how much fear or anxiety they would feel on a scale from (0) none to (3) severe and how likely they would be to avoid from never (0%) to usually (67–100%). Example items include: Talking to people in authority, Looking at people you don’t know very well in the eyes, and Giving a report to a group. A total score was computed by summing all items together. Higher scores represent higher levels of social anxiety. The scale exhibited good internal reliability (α = .94). Supplementary Figure S5 displays the distributions of social anxiety scores (*Min* = 16; *Max* = 88). 12 out of 34 participants had scores higher than 60, a commonly cited cutoff score for social anxiety (Mennin et al., 2002).

##### 2.6.2.2 Social interaction quality

Participant impressions of the social interaction quality were assessed via the social interaction quality questionnaire, adapted from Berry and Hansen (1996). Participants were instructed: “Thinking about the interaction you just had, please answer the following questions.” There were 11 items answered on an 8-point scale from (1) not at all to (8) very much. Example questions include: The conversation was smooth, natural, and relaxed. My friend disclosed a lot to me; and I influenced the conversation. I enjoyed the conversation. Items were averaged together to create a composite score where higher scores represent more positive impressions of the social interaction quality. Participants completed the scale after the social interaction during Visit 1 and after the scanner task during Visit 2. The scale exhibited good internal reliability at both assessments (α*’s* ≥ .75).

##### 2.6.2.3 Friendship quality

Participant impressions of friendship quality were assessed via the Respondents Affection section (16 items) of The McGill Friendship Questionnaires (Mendelson & Aboud, 1999). Participants were instructed, “The items on this form concern your feelings for the friend you just interacted with. Imagine that the blank space in each item contains your friend’s name. With him or her in mind, decide how much you agree or disagree with the item. There are no right or wrong answers, because adults’ feelings for friends differ from person to person. Just honestly describe your feelings for your friend. All questions can be answered on a 9-point scale from (-4) very much disagree to (4) very much agree.” A single score was created for the Respondents Affection section by averaging all items together. The questionnaire exhibited good internal reliability (α = .95).

##### 2.6.2.4 Discomfort watching self

After completing the scanner task, participants were asked “How uncomfortable were you watching yourself?” Participants responded on a 10-point scale from 1 – not uncomfortable to 10 – very uncomfortable.

### 2.7 Data analysis plan

To assess relations between inter-brain neural similarity, perspective condition (Same-Stimuli, Self-Perspective, Friend Perspective), affective similarity and social anxiety symptoms, we conducted a multilevel model in R using the lme4 package (Bates et al., 2015). Inter-brain neural similarity was entered as a continuous outcome variable. Mentalizing region (dmPFC – 0, vmPFC – 1, rTPJ – 3, lTPJ – 4) and perspective condition (Same-Stimuli – 0, Self-Perspective – 1, Friend-Perspective – 2) were entered as categorical predictor variables. Trait affective similarity, state affective similarity, and social anxiety symptoms were entered as continuous predictor variables. State affective similarity was also entered as a random effect. Interactions between perspective condition, state affective similarity, and social anxiety symptoms were included. The social anxiety symptoms variable was centered before being included as an interaction term. The model was nested by dyad. Time was not included in the model because the order of trials was different within dyads for the Self-Perspective and Friend-Perspective conditions. Missing data (Supplementary Section S5) for affective ratings were handled using multiple imputation in R implemented using the mice package (van Buuren & Groothuis-Oudshoorn, 2011). The model was assessed over the pooled results of 25 data sets (see Supplementary Section S6 for additional detail). While we were well powered to assess within-person variation (the relation between inter-brain neural similarity and affective similarity), we were less powered to consider between-person variation (social anxiety symptoms as a moderator), supporting future hypothesis generation.

We conducted additional sensitivity analyses. First, we assessed the model over the listwise deleted data. Second, we ran a model controlling for self-reported discomfort watching self, social interaction quality at both assessments, and friendship quality. Third, we examined the model without including social anxiety symptoms. Fourth, we examined a potential Perspective Condition x Mentalizing Region effect. Finally, we examined the effects of perspective condition, affective similarity, and social anxiety symptoms on inter-brain neural similarity within individual mentalizing regions.

## 3 Results

Table 2 displays descriptive statistics and correlations for between-subjects variables. Our primary model (Table 3) suggested that greater affective similarity was related to greater inter-brain neural similarity when participants both viewed the social interaction from their own perspective (Self-Perspective condition; how they experienced the live social interaction) compared to when participants viewed identical visual stimuli (Same-Stimuli condition). Figure 4 displays the two-way interaction between State Affective Similarity and the Self-Perspective condition (*b* = 0.023, *p* = .015). On trials that dyads experienced more affective similarity than they did on average, they exhibited greater inter-brain neural similarity during the Self-Perspective condition. Additionally, social anxiety symptoms were related to differences in the affective similarity-inter-brain neural similarity relation when participants both viewed the social interaction from their friend’s perspective (Friend-Perspective condition) compared to when participants viewed identical visual stimuli (Same-Stimuli condition). Figure 5 displays the three-way interaction between State Affective Similarity, the Friend-Perspective condition, and Social Anxiety Symptoms (*b* = -0.001, *p* = .013).

**Fig. 4.**
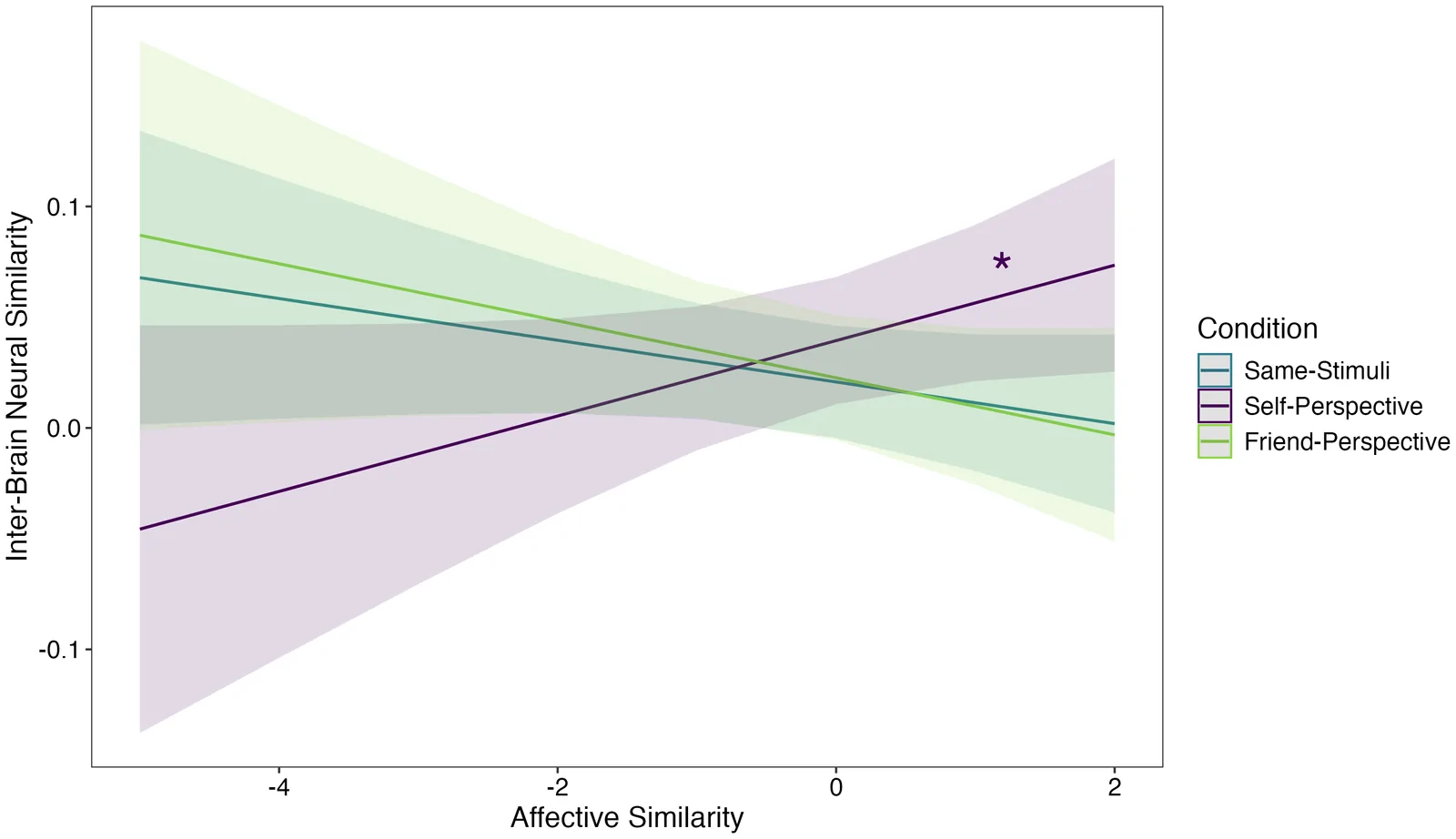
In our model, the Same-Stimuli Condition (in blue) was our reference condition and the Self-Perspective Condition (in purple) was significantly different from the reference condition. The Friend-Perspective Condition (in green) was not significantly different from the reference condition. Thus, when dyads expressed more affective similarity, they also showed more inter-brain neural similarity specifically when both participants viewed the social interaction from their own perspective (Self-Perspective condition) in comparison to the Same-Stimuli condition. Significant simple slopes test indicated by asterisks (Supplementary Table S13).

**Fig. 5.**
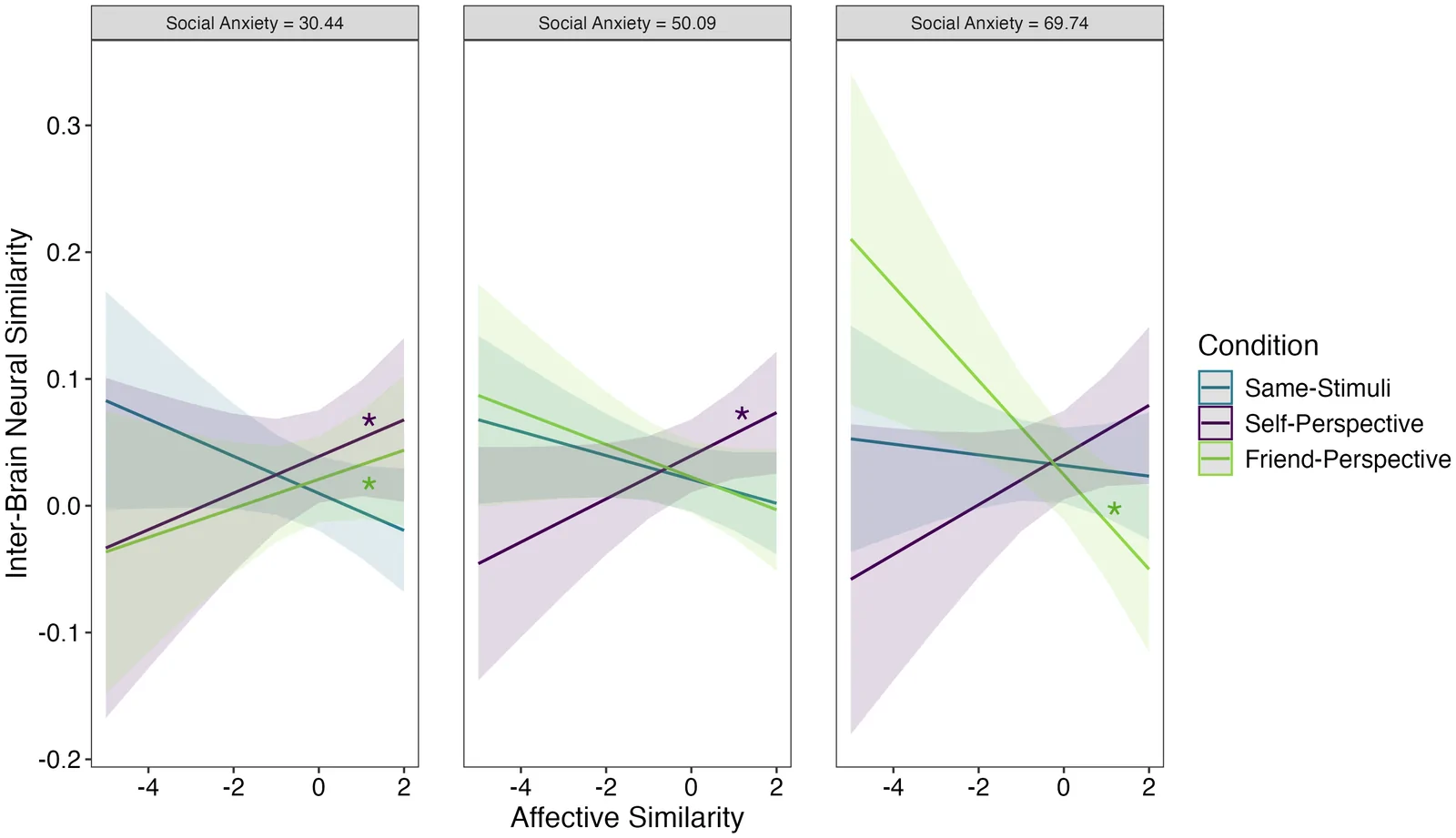
In our model, the Same-Stimuli Condition (in blue) was our reference condition and the Friend-Perspective condition (in green) was significantly different from the reference condition as a function of social anxiety symptoms. For participants with fewer social anxiety symptoms, for both the Self-Perspective condition (in purple) and the Friend-Perspective condition, when dyads expressed more affective similarity, they also showed more inter-brain neural similarity. In contrast, for participants with greater social anxiety symptoms, for the Friend-Perspective condition, when dyads expressed less affective similarity, they showed more inter-brain neural similarity, despite showing the same effect for the Self-Perspective condition as the sample on the whole. Significant simple slopes test indicated by asterisks (Supplementary Table S14).

**Table 2.** Zero-order correlations and descriptive statistics for between-subjects variables.

	2.	3.	4.	5.	*M*	*SD*
1. Social anxiety	-.08	-.08	.14	.33	48.85	21.40
2. Social interaction quality visit 1		**.75**	.33	.08	6.67	0.75
3. Social interaction quality visit 2			**.39**	.23	6.52	0.85
4. Friendship quality				.07	3.62	0.70
5. Discomfort watching self during BFFs task					5.76	2.65

*Note*: N = 34. *M* = Mean; *SD* = Standard Deviation; bolding represents *p* < .05.

**Table 3.** Multilevel model assessing contributions of condition, affective similarity, and social anxiety symptoms to inter-brain neural similarity.

	Est	Std. Est	SE	t	*p*
Fixed effects					
Intercept	0.008	-0.021	0.041	0.20	.842
Trait affective similarity	-0.012	-0.009	0.029	-0.42	.675
vmPFC	0.010	0.026	0.011	0.89	.373
rTPJ	0.002	0.005	0.011	0.15	.878
lTPJ	0.014	0.035	0.011	1.18	.237
State affective similarity	-0.008	-0.025	0.005	-1.66	.097
Self-perspective	0.007	0.016	0.010	0.71	.475
Friend-perspective	0.000	-0.001	0.010	0.01	.989
Social anxiety	0.001	0.028	0.000	1.47	.141
Affective similarity x self-perspective	0.023	0.070	0.009	2.45	**.015**
Affective similarity x friend-perspective	-0.001	-0.008	0.009	-0.06	.949
Affective similarity x social anxiety	0.000	0.012	0.000	0.84	.399
Self-perspective x social anxiety	-0.001	-0.040	0.001	-1.43	.153
Friend-perspective x social anxiety	-0.000	-0.019	0.000	-0.81	.421
Affective similarity x self-perspective x social anxiety	-0.000	-0.003	0.000	-0.18	.858
Affective similarity x friend-perspective x social anxiety	-0.001	-0.072	0.000	-2.49	**.013**
Random effects					
Dyad – intercept	0.001 (0.032)				
Dyad – affective similarity	0.0002 (0.016)				

*Note:* Pooled results across 25 imputed data sets with 9072 observations each; bolding represents *p* < .05.

During the Friend-Perspective condition, reporting fewer social anxiety symptoms was associated with greater inter-brain neural similarity on trials that dyads experienced more affective similarity than they did on average. Conversely, reporting more social anxiety symptoms was associated with greater inter-brain neural similarity on trials that dyads experienced less affective similarity than they did on average.

### 3.1 Sensitivity analyses

The model reported above was consistent with models assessed with control variables (social interaction quality at both assessments, friendship quality, discomfort watching self; Supplementary Table S2), excluding interactions with social anxiety symptoms (Supplementary Table S3) and with listwise deleted data (Supplementary Table S4). In the primary model (Table 3), inter-brain neural similarity was similar across all examined mentalizing regions (no direct effects of region on inter-brain neural similarity). However, we wanted to explore potential differences by region. A model assessing a potential Perspective Condition x Mentalizing Region interaction on inter-brain neural similarity found no effect (Supplementary Table S5), again suggesting that inter-brain neural similarity was similar across mentalizing regions regardless of perspective condition (Same-Stimuli, Self-Perspective, Friend-Perspective). We ran models assessing relations between inter-brain neural similarity, perspective condition, affective similarity, and social anxiety symptoms in the individual regions (Supplementary Table S6 and Supplementary Table S7; Fig. 6). Within the dmPFC, greater affective similarity was related to greater inter-brain neural similarity when both participants viewed the social interaction from their own perspective (*b* = 0.036, *p* = .043), in keeping with the primary model. Within the rTPJ, more social anxiety symptoms were related to less inter-brain neural similarity when both participants viewed the social interaction from their friend’s perspective (*b* = -0.002, *p* = .033), a finding that did not emerge in the primary model. No effects were found for inter-brain neural similarity within the vmPFC or the lTPJ. The three-way interaction observed in the primary model did not reach significance in individual regions.

**Fig. 6.**
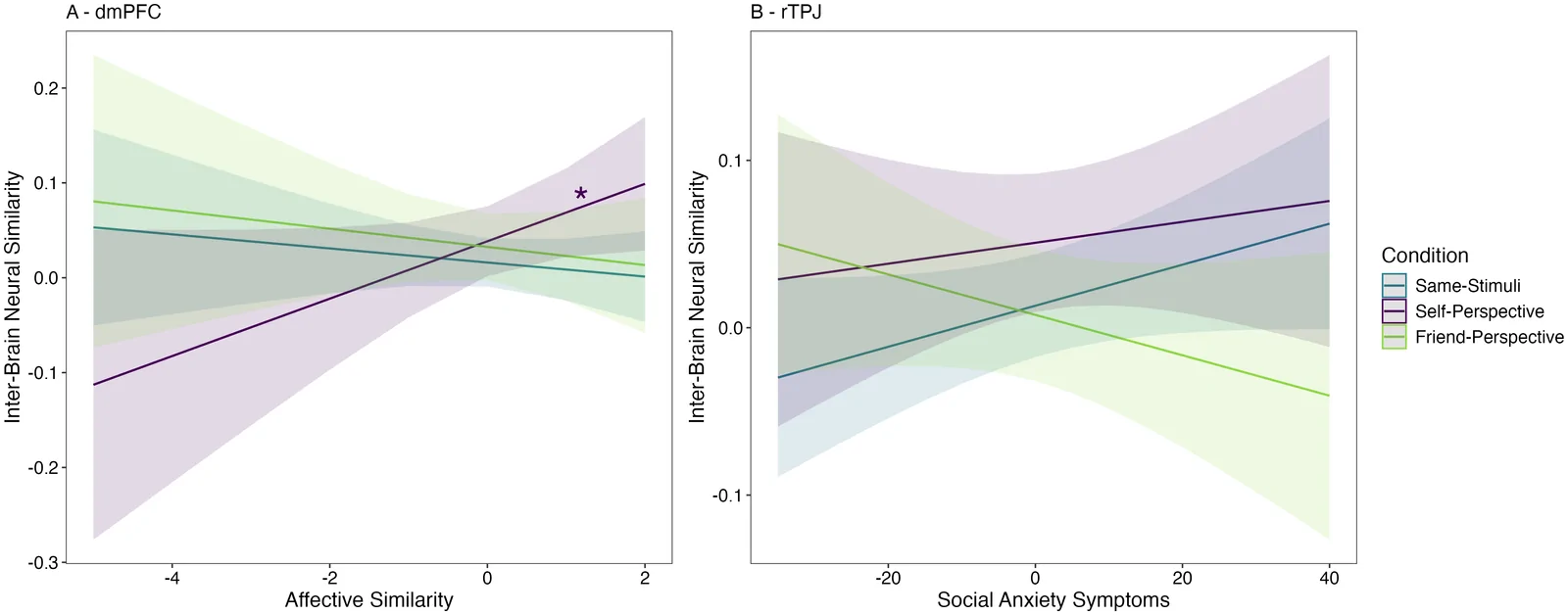
Effects on inter-brain neural similarity within specific mentalizing regions again with the Same-Stimuli condition as the reference condition. (A - dmPFC) When affective similarity was greater than average, inter-brain neural similarity within the dmPFC was greater when both participants viewed the social interaction from their own perspective (Self-Perspective condition). (B – rTPJ) Higher levels of social anxiety symptoms related to less inter-brain neural similarity within the rTPJ when both participants viewed the social interaction from their friend’s perspective (Friend-Perspective condition). Significant simple slopes test indicated by asterisks (Supplementary Table S15 & Supplementary Table S16).

Additionally, we conducted the same models in a Bayesian framework using brms (Bürkner, 2021) in R. Bayesian models can be better for modeling small N data sets as in the current paper. Supplementary Tables S8–S12 display the results of the Bayesian models which reflect the similar results as obtained in the frequentist models, lending additional support to the findings.

## 4 Discussion

The current study assessed contributions of perspective, affective similarity, and social anxiety symptoms to inter-brain neural similarity in mentalizing regions within friend pairs. To do so, we compared inter-brain neural similarity when visual stimuli were identical (Same-Stimuli) against inter-brain neural similarity when visual stimuli were not identical and instead matched in perspective (Self-Perspective and Friend-Perspective). We found evidence that affective similarity was related to inter-brain neural similarity when friends took the same perspective (Self-Perspective and Friend-Perspective) rather than when visual stimuli were identical (Same-Stimuli). When both participants viewed the social interaction from their own perspective (Self-Perspective condition), greater affective similarity was associated with greater inter-brain neural similarity. Additionally, when both participants viewed the social interaction from their friend’s perspective (Friend-Perspective condition), participants lower in social anxiety symptoms exhibited greater inter-brain neural similarity in the context of greater affective similarity, matching the same pattern of results observed during the Self-Perspective condition. Conversely, participants higher in social anxiety symptoms exhibited greater inter-brain neural similarity in the context of less affective similarity, in contrast to the pattern of results observed during the Self-Perspective condition. The current results suggest affective similarity may coordinate shared representations of social experience, particularly when both participants are experiencing a social interaction from their own perspective, as they would in everyday life. However, social anxiety symptoms may alter relations between perspective and affective similarity, particularly when taking an interaction partner’s perspective and viewing oneself.

Natural social interactions are dynamic and recruit the mentalizing network differently than do non-social stimuli (Deuse et al., 2016) and non-social interactions (Alkire et al., 2018; Rice & Redcay, 2016). Natural social interactions are also the context in which mentalizing abilities are most valuable and the context in which social anxiety is most likely to cause difficulties (Jarcho & Guyer, 2018). Our naturalistic neuroscience paradigm capitalized on using participants’ real-life social interactions to examine inter-brain neural similarity within the mentalizing network. This greater degree of ecological validity made the stimuli highly relevant to each participant and provided an opportunity to compare classic conceptualizations of inter-brain neural similarity against inter-brain neural similarity within social interaction perspectives.

Inter-brain neural similarity is typically measured by having participants watch identical stimuli in the form of a movie (Dmochowski et al., 2014), similar to our Same-Stimuli condition in which visual stimuli were identical. Although inter-brain neural similarity when watching movies has been related to similarity in affective responses (Nummenmaa et al., 2012), social interaction quality (Dziura et al., 2023), and friendship quality (Parkinson et al., 2018), having similar neural responses to a movie is different than creating a shared representation of a lived social interaction. Our paradigm suggested that greater affective similarity might particularly enhance inter-brain neural similarity when both participants experience a social interaction from their own perspective, aiding people in building a shared representation of their social experience. Socially, this indicates the importance of affective similarity specifically in interactive context and not solely in creating shared meaning about observed stimuli in general.

However, it was the Friend-Perspective condition, rather than the Self-Perspective condition, that drew out individual differences related to social anxiety symptoms. The Friend-Perspective condition, while naturalistic, is not how people would experience a social interaction in everyday life. Interestingly, participants low in social anxiety symptoms exhibited greater inter-brain neural similarity in the context of greater affective similarity both when viewing the interaction from their own view (Self-Perspective condition) and their friend’s view (Friend-Perspective condition). Participants higher in social anxiety symptoms exhibited the opposite relation. Research suggests people represent themselves and close others similarly in the mPFC, but that lonely individuals show less similarity in neural representations of themselves (Courtney & Meyer, 2020). Potentially, participants lower in social anxiety symptoms more readily integrated the experience of watching the social interaction from their own or their friend’s view more similarly than participants higher in social anxiety symptoms. In this way, having participants view a social interaction from an unfamiliar perspective may be more sensitive to individual differences than simply having participants “re-live” the social interaction solely from their own perspective. Future work could probe the subsequent cognitive and socioemotional processes triggered by manipulating our typical social perspective.

Mentalizing abilities and the mentalizing network support the ability to recognize the intentions and emotions of others (Frith & Frith, 2003). However, evidence also suggests mentalizing network regions are associated with self versus other distinctions (Denny et al., 2012; Saxe, Moran, et al., 2006), with the mPFC being more attuned to self-representations (and the TPJ being more attuned to other-representations). In our primary model, we found inter-brain neural similarity was consistent across mentalizing regions. Additionally, in one of our sensitivity analyses we found no interaction between perspective condition and mentalizing region on inter-brain neural similarity. Some evidence suggests that different mentalizing regions may play an important role in self vs. other distinctions (Denny et al., 2012; Qin & Northoff, 2011). However, in our models assessing mentalizing regions simultaneously, we found no such distinction. Our results could be due to the nature of the task which likely caused participants to consider both their own and their friend’s mental states throughout each clip. This is likely how natural social interactions function with constant updating of both self and other relevant information.

When assessing relations between perspective, affective similarity, and social anxiety symptoms in individual regions, we found some evidence for possible regional differences. When both participants viewed the social interaction from their own perspective (Self-Perspective condition), greater affective similarity was associated with greater dmPFC inter-brain neural similarity. Additionally, when both participants viewed the social interaction from their friend’s perspective (Friend-Perspective condition), having more social anxiety symptoms was related to less rTPJ inter-brain neural similarity. No significant effects emerged for either the vmPFC or the lTPJ. These results suggest some specificity in self versus other distinctions could exist by mentalizing region. As anticipated, an mPFC region (dmPFC) appeared more relevant for relations during the Self-Perspective condition and a TPJ region (rTPJ) appeared more relevant for relations during the Friend-Perspective condition. Of note, the relation between social anxiety symptoms and inter-brain neural similarity was observed only when modeling the rTPJ independently and not in the context of the larger model.

Because evidence suggests the vmPFC is particularly engaged in affective mentalizing or tracking emotion cues (Arioli et al., 2021), it is perhaps surprising that significant effects were not detected for the vmPFC. However, research on the vmPFC has been mixed, potentially because the vmPFC likely exhibits diverse functionality associated with particular organization, including distinct social, emotion, and valuation domains (Chen et al., 2026). Future work could examine if focusing on specific organization within the vmPFC reveals patterns similar to the patterns observed in the dmPFC. The rTPJ has long been considered central to representing other’s internal states and recent work supports this conclusion (Golec-Staśkiewicz et al., 2022). However, the same study proposed the lTPJ may be involved in detecting discrepancies in beliefs, such as in a classic belief-reasoning task. The current study was not designed to tap into clear differences in belief-reasoning and rather focused on letting friends naturalistically engage with one another. This could be one reason significant results were not seen within the lTPJ. However, future work could naturalistically assess belief-reasoning during fMRI data collection to assess this possibility further. Given the novel nature of the study, it will be important to examine the possibilities and replicate the significant findings in larger samples.

Previous research is mixed regarding relations between mentalizing abilities and social anxiety. Some evidence suggests socially anxious people engage in too much self-focused thought (Boehme et al., 2015), while other evidence suggests they engage in over-mentalizing (Ballespí et al., 2019; Washburn et al., 2016). The current paradigm cannot necessarily distinguish between these two potential mechanisms. For example, in the Friend-Perspective condition, both members of a dyad saw themselves in the clips which could lead to increased self-focused thought. However, we did control for discomfort while watching the self and our results remained consistent. Additionally, we found that when both participants viewed the social interaction from their friend’s perspective (Friend-Perspective condition), reporting more social anxiety symptoms was related to less inter-brain neural similarity within the rTPJ. As the rTPJ is strongly associated with representing others (Denny et al., 2012; Saxe, Moran, et al., 2006), our result might point to over-mentalizing rather than self-focused thought. However, we stress these results are preliminary and more work is needed to clearly differentiate these potential mechanisms. One potential method could be having participants thoroughly describe what they were thinking about during each clip, rather than only reporting on how positively and negatively they felt about each clip. Detailed descriptions from participants could be coded for self-focused versus other-focused language and could help distinguish between these theories.

We also found that when both participants viewed the social interaction from their friend’s perspective (Friend-Perspective condition), more social anxiety symptoms were related to greater inter-brain neural similarity when there was less affective similarity. Potentially, more socially anxious participants could sense a discrepancy in affective state and worked harder to interpret their friend’s experience to try to create a shared understanding of the interaction (Baek & Parkinson, 2022; Lahnakoski et al., 2014). Given the small sample size for detecting between-subjects effects, our results provide an excellent foundation for developing future naturalistic paradigms that could better untangle the experience of mentalizing for socially anxious individuals during social interactions.

Limitations of the current study include the small sample size. Although the number of trials provided good power to detect within-person effects, between-subjects effects, including effects associated with social anxiety symptoms, should be considered as early evidence that will need to be replicated in future work in larger sample sizes. However, our sensitivity analyses, including the Bayesian models, do point to the potential robustness of these relations. Additionally, generalizability is impacted by the sample demographics given that all participants were college students, and the majority were White. Future work should replicate these findings in more diverse samples.

The current analyses specifically focused on regions within the mentalizing network. However, emotion processing is likely an important component underlying how people, and perhaps especially more socially anxious people, respond to previously lived social interactions. Future work should examine the role of emotion processing neural regions and potential interactions between emotion processing regions and the mentalizing network to better understand the scope of differences in socioemotional experience.

Additionally, within the mentalizing network we chose specifically to focus on the mPFC and TPJ as previous research suggests they are closely associated with distinctions between thinking about the self (Courtney & Meyer, 2020; Qin & Northoff, 2011) and others (Denny et al., 2012; Saxe, Moran, et al., 2006). However, our whole-brain analyses did suggest additional overlap between signal during our task and the Neurosynth mentalizing map in the precuneus and cerebellar regions. We chose not to focus on these regions as we did not have specific hypotheses about how they would relate to thinking about the self versus thinking about a friend. Future work should more deeply assess the role these additional regions might play. For example, a body of work is now identifying the critical role the cerebellum plays in socio-emotional experience and learning (Van Overwalle, 2024). Similarly, the precuneus may be particularly relevant for tracking others beliefs (Arioli et al., 2023). The current task was not explicitly designed to assess learning or tracking beliefs. However, a similar naturalistic design could be employed to get at these important questions.

We have framed our results as marking how dyads developed shared representations of the experience. However, this is a broad statement and there is potential for nuance. For example, dyads may have found common positive representations, such as shared comfort and understanding, or less positive representations, such as confusion or avoidance. Further, shared representations could vary across clips and between people higher and lower in social anxiety symptoms. Future work could more closely examine these possible relations.

Importantly, in the design of this study, we weighed several potential tradeoffs between experimental control and ecological validity. First, we designed the scanner task so that the first run was always the Self-Perspective Run and the second run was always the Friend-Perspective Run (Fig. 1B). We opted not to counterbalance as we hypothesized that participants may feel discomfort from viewing themselves in the Friend-Perspective Run and we did not want this discomfort to influence responses to the Self-Perspective Run. Thus, it is possible there could be confounding effects such as fatigue or habituation. However, 1) the entire task is a repetition of a social interaction the friends already shared and 2) in our previous work using this task, we showed that mentalizing network connectivity did not differ between the runs and rather varied as a function of affective context (Vallorani et al., 2026), suggesting the order of the runs may not have significantly impacted results. Second, because of the naturalistic design, it was impossible to ensure that every dyad was exposed to the exact same amount of positive and negative affect across the clips used in the scanner task. We address this concern by using participants’ own subjective ratings of each clip and provide evidence that participants themselves viewed negative clips as more negative and positive clips as more positive than neutral clips (Supplementary Section S3).

Evidence suggests the ways people attend to real-world experiences (Risko et al., 2016) and neurally process ostensibly real-world social interactions (Alkire et al., 2018; Rice & Redcay, 2016) are different from how people respond to traditional, third-person, static paradigms. Additionally, recent evidence suggests naturalistic movie viewing outperforms simple resting-state tasks in terms of predicting participant phenotypes (Finn & Bandettini, 2021). Naturalistic data afford the opportunity to examine how people experience the world and people around them. For example, we found affective similarity related to neural similarity specifically when re-living the social interaction as they would have originally experienced it, from their own perspective. Collecting data from people’s unique, idiosyncratic experiences and modeling those data continuously can elucidate nuance that might be lost in averaged, third-person paradigms. Such paradigms might be particularly important for understanding disorders associated with difficulties during social interactions such as social anxiety or autism spectrum disorder. If the interaction context is the source of difficulty, attempting to understand social processing outside the interaction context is necessarily limiting. Given the importance of evaluating processes within their naturalistic contexts, we believe both tradeoffs we made between ecological validity and experimental control are worthwhile.

However, we note that the study did not directly contrast a third-person paradigm with our naturalistic paradigm. Previous work has made such direct contrasts in relation to social attention research (Fu et al., 2019). It is important for future work to similarly directly test the same participants using both third-person and naturalistic neuroscience paradigms to highlight the benefits of both approaches. Further, we suggest iterating on such naturalistic paradigms to find the best balance between ecological validity and experimental control to best understand individual variation within the social interaction context.

In conclusion, we designed and implemented a naturalistic neuroscience paradigm to investigate inter-brain neural similarity within the mentalizing network across friend pairs. We found that when both participants in a dyad viewed the social interaction from their own perspective (Self-Perspective condition), greater affective similarity than their average was associated with greater inter-brain neural similarity. Additionally, when both participants in a dyad viewed the social interaction from their friend’s perspective (Friend-Perspective condition), participants who reported fewer social anxiety symptoms showed the same pattern as above (greater affective similarity related to greater inter-brain neural similarity). Conversely, for participants who reported more social anxiety symptoms, less affective similarity was related to greater inter-brain neural similarity. The current results suggest the utility of naturalistic neuroscience paradigms to investigate socioemotional processing within the mentalizing network and provide evidence for future hypothesis generation, particularly regarding the roles of affect and social anxiety in creating shared meaning during natural social interactions.

## Supplementary Material

Supplementary Material

## Data Availability

Data (Vallorani et al., 2023) and analytic code (Vallorani, 2024) are publicly available. The analyses were not preregistered.
