# Supplementary material for: Mentalizing from self and friend perspectives: Relations between inter-brain neural similarity, affective similarity and social anxiety symptoms
Source: Imaging Neurosci (Camb). 2026 Oct 16;4:IMAG.a.1403. doi: 10.1162/IMAG.a.1403 (PMC13681053; doi:10.1162/IMAG.a.1403)
Supplement: Supplementary file 1 [file IMAG.a.1403_supp.pdf]

Supplement

S1. Inclusion criteria

Inclusion criteria were that participants be between the ages of 18 and 25, fluent in English, have 20/40 vision or vision that could be corrected to at least 20/40 with contacts (not glasses), be eligible to participate in an MRI scan (i.e., no contraindications such as weight greater than 250 lbs or braces) and be vaccinated against Covid-19. Participants were excluded if they were on any medications that might impact blood flow or cause drowsiness, experiencing any conditions that might cause atypical eye movements, or had a self-reported history of any of the following: Psychotic Disorder, Bipolar 1 Disorder, Autism Spectrum Disorder, Reactive Attachment Disorder, Pervasive Developmental Disorder, Motor Disorder, Intellectual Disability, Neurological Disorder or head injury with loss of consciousness for greater than 5 minutes.

S2. Excluded participants

Dyads were excluded when one or more of the participants in the dyad was missing fMRI data as this kept us from computing neural similarity for the dyad. For 7 dyads, both participants were missing fMRI data (3 dyads did not have enough affective variability to create stimuli, 1 dyad was collected on an older MET system and the videos were not comparable to later stimuli, 1 dyad did not follow instructions during the social interaction, 1 dyad the room view camera crashed and data could not be behaviorally coded, 1 dyad 1 participant had an undisclosed piercing and the other participant decided not to complete visit 2). For 9 dyads, one of the two participants were missing fMRI data (3 sound failed during the runs, 2 decided not to complete the

visit, 2 moved too much during the runs, 1 head did not fit in the coil, 1 had an undisclosed piercing).

S3. Pupil invisible system and stimuli creation

Each mobile eye-tracker includes both eye cameras and a scene camera. Videos used for stimuli were extracted from the scene camera which had a square aspect ratio and a field of view of 1088 X 1080 pixels. Video was collected using the Pupil Invisible Application on a OnePlus 8 Android phone. Data collection was monitored using Pupil Monitor which projected the videos being collected through the Pupil Invisible Application to a screen the researchers could watch, allowing the researchers to ensure data quality.

Third-person videos of the social interaction were synced with the individual perspective videos in Pupil Player Version 3.5.7. Post syncing, the individual perspective videos and the third-person videos were exported separately (ensuring that timing across all videos was exact). The third-person videos, with individual perspective videos embedded, were coded at a broad level for negative and positive affect. First, the 20-minute social interaction video was read into Databrary version 1.5.3. The video was then chunked into 20-second epochs for coding.

Each participant was coded separately for any presence of positive affect and any presence of negative affect based on facial expressions (smiling/frowning), vocal expressions (laughter/groaning) and vocal tone for each epoch. Thus, for each epoch there were 4 potential codes: participant 1 negative affect (0/1), participant 2 negative affect (0/1), participant 1 positive affect (0/1) and participant 2 positive affect (0/1),

where 1 is present and 0 is not present. Epochs in which a researcher's voice could be heard providing instructions to participants were excluded from coding.

Post coding, in-house R scripts were used to select epochs to represent moments of negative, neutral and positive affect. First, dyadic mean negative and mean positive affect were computed for each epoch. A *selected affect score* was computed via the following: Epochs with mean negative affect > 0.5 and mean positive affect ≤ 0.5 were considered negative. Epochs with mean negative affect ≤ 0.5 and mean positive affect > 0.5 were considered positive. Epochs with mean negative affect equaling 0 and mean positive affect ≤ 0.5 or mean negative affect ≤ 0.5 and mean positive affect equaling 0 were considered neutral. Additionally, a *relative affect score* was computed by subtracting mean negative affect from mean positive affect.

Epochs were selected for consideration as stimuli when the *selected affect score* matched the *relative affect score* or when the *selected affect score* equaled 0 and the *relative affect score* equaled ±0.5. Of epochs that were selected for consideration, R scripts then randomly selected 6 negative, 6 neutral and 6 positive epochs. These epochs were then randomized so that epochs of the same affect would never be presented back-to-back during the scanner task. The timing for each of the clips was extracted. The perspective videos were imported into Final Cut Pro Version 10.6.4 and 20-second clips from each participant's perspective of the selected epochs were exported as stimuli.

Participants rated clips we selected as positive as more positive than clips we selected as neutral ($b = -0.57$, $p = .011$) and rated clips we selected as negative as more negative than clips we selected as neutral ($b = 0.99$, $p < .001$).

S4. fMRIPrep

Preprocessing of B0 inhomogeneity mappings. A B0-nonuniformity map (or fieldmap) was estimated based on two (or more) echo-planar imaging (EPI) references with topup (Andersson et al., 2003; FSL 6.0.5.1:57b01774).

Anatomical data preprocessing. A total of 1 T1-weighted (T1w) images were found within the input BIDS dataset.The T1-weighted (T1w) image was corrected for intensity non-uniformity (INU) with N4BiasFieldCorrection (Tustison et al., 2010), distributed with ANTs 2.3.3 (Avants et al., 2008; RRID:SCR_004757), and used as T1w-reference throughout the workflow. The T1w-reference was then skull-stripped with a Nipype implementation of the antsBrainExtraction.sh workflow (from ANTs), using OASIS30ANTs as target template. Brain tissue segmentation of cerebrospinal fluid (CSF), white-matter (WM) and gray-matter (GM) was performed on the brain-extracted T1w using fast (Zhang et al., 2001; FSL 6.0.5.1:57b01774, RRID:SCR_002823). Brain surfaces were reconstructed using recon-all (Dale et al., 1999; FreeSurfer 7.3.2, RRID:SCR_001847), and the brain mask estimated previously was refined with a custom variation of the method to reconcile ANTs-derived and FreeSurfer-derived segmentations of the cortical gray-matter of Mindboggle (Klein et al., 2017; RRID:SCR_002438). Volume-based spatial normalization to two standard spaces (MNI152NLin2009cAsym, MNI152NLin6Asym) was performed through nonlinear registration with antsRegistration (ANTs 2.3.3), using brain-extracted versions of both T1w reference and the T1w template. The following templates were were selected for spatial normalization and accessed with TemplateFlow (Ciric et al., 2022; 23.0.0): ICBM 152 Nonlinear Asymmetrical template version 2009c (Fonov et al., 2009 ;

RRID:SCR_008796; TemplateFlow ID: MNI152NLin2009cAsym], FSL\u2019s MNI ICBM 152 non-linear 6th Generation Asymmetric Average Brain Stereotaxic Registration Model [Evans et al. (2012), RRID:SCR_002823; TemplateFlow ID: MNI152NLin6Asym).

Functional data preprocessing. For each of the 3 BOLD runs found per subject (across all tasks and sessions), the following preprocessing was performed. First, a reference volume and its skull-stripped version were generated by aligning and averaging 1 single-band references (SBRefs). Head-motion parameters with respect to the BOLD reference (transformation matrices, and six corresponding rotation and translation parameters) are estimated before any spatiotemporal filtering using mcflirt (FSL 6.0.5.1:57b01774, Jenkinson et al. 2002). The estimated fieldmap was then aligned with rigid-registration to the target EPI (echo-planar imaging) reference run. The field coefficients were mapped on to the reference EPI using the transform. BOLD runs were slice-time corrected to 0.274s (0.5 of slice acquisition range 0s-0.547s) using 3dTshift from AFNI (Cox and Hyde 1997, RRID:SCR_005927). The BOLD reference was then co-registered to the T1w reference using bbregister (FreeSurfer) which implements boundary-based registration (Greve & Fischl, 2009). Co-registration was configured with six degrees of freedom. First, a reference volume and its skull-stripped version were generated using a custom methodology of fMRIPrep. Several confounding time-series were calculated based on the preprocessed BOLD: framewise displacement (FD), DVARS and three region-wise global signals. FD was computed using two formulations following Power (absolute sum of relative motions, Power et al. (2014)) and Jenkinson (relative root mean square displacement between affines, Jenkinson et al.

(2002)). FD and DVARS are calculated for each functional run, both using their implementations in Nipype (following the definitions by Power et al. 2014). The three global signals are extracted within the CSF, the WM, and the whole-brain masks. Additionally, a set of physiological regressors were extracted to allow for component-based noise correction (Behzadi et al., 2007; CompCor). Principal components are estimated after high-pass filtering the preprocessed BOLD time-series (using a discrete cosine filter with 128s cut-off) for the two CompCor variants: temporal (tCompCor) and anatomical (aCompCor). tCompCor components are then calculated from the top 2% variable voxels within the brain mask. For aCompCor, three probabilistic masks (CSF, WM and combined CSF+WM) are generated in anatomical space. The implementation differs from that of Behzadi et al. in that instead of eroding the masks by 2 pixels on BOLD space, a mask of pixels that likely contain a volume fraction of GM is subtracted from the aCompCor masks. This mask is obtained by dilating a GM mask extracted from the FreeSurfer\u2019s aseg segmentation, and it ensures components are not extracted from voxels containing a minimal fraction of GM. Finally, these masks are resampled into BOLD space and binarized by thresholding at 0.99 (as in the original implementation). Components are also calculated separately within the WM and CSF masks. For each CompCor decomposition, the k components with the largest singular values are retained, such that the retained components\u2019 time series are sufficient to explain 50 percent of variance across the nuisance mask (CSF, WM, combined, or temporal). The remaining components are dropped from consideration. The head-motion estimates calculated in the correction step were also placed within the corresponding confounds file. The confound time series derived from head motion

estimates and global signals were expanded with the inclusion of temporal derivatives and quadratic terms for each (Satterthwaite et al., 2013). Frames that exceeded a threshold of 0.5 mm FD or 1.5 standardized DVARS were annotated as motion outliers. Additional nuisance timeseries are calculated by means of principal components analysis of the signal found within a thin band (crown) of voxels around the edge of the brain, as proposed by (Patriat et al., 2017). The BOLD time-series were resampled into standard space, generating a preprocessed BOLD run in MNI152NLin2009cAsym space. First, a reference volume and its skull-stripped version were generated using a custom methodology of fMRIPrep. Automatic removal of motion artifacts using independent component analysis (Pruim et al., 2015; ICA-AROMA) was performed on the preprocessed BOLD on MNI space time-series after removal of non-steady state volumes and spatial smoothing with an isotropic, Gaussian kernel of 6mm FWHM (full-width half-maximum). Corresponding \u201cnon-aggresively\u201d denoised runs were produced after such smoothing. Additionally, the \u201caggressive\u201d noise-regressors were collected and placed in the corresponding confounds file. All resamplings can be performed with a single interpolation step by composing all the pertinent transformations (i.e. head-motion transform matrices, susceptibility distortion correction when available, and co-registrations to anatomical and output spaces). Gridded (volumetric) resamplings were performed using antsApplyTransforms (ANTs), configured with Lanczos interpolation to minimize the smoothing effects of other kernels (Lanczos, 1964). Non-gridded (surface) resamplings were performed using mri_vol2surf (FreeSurfer).

Many internal operations of fMRIPrep use Nilearn 0.9.1 (Abraham et al., 2014; RRID:SCR_001362), mostly within the functional processing workflow. For more details of the pipeline, see the section corresponding to workflows in fMRIPrep\u2019s documentation.

S5. Missing Data

2 dyads were missing a run of fMRI data. For one dyad, this was because one participant lost sound during only the self run. For the second dyad, there was a malfunction with one of the MET glasses causing only one participant's view to be recorded, making it so one participant only had the self run and the other participant only had the friend run. Multiple participants were missing some responses about how they felt after a clip during the scanner task.

S6. Multiple Imputation

Because we believe listwise deletion of data is more biasing than handling missing data, we used multiple imputation. A post-hoc MCAR Little's test suggested the data were not missing completely at random. This is not surprising given the repeated nature of the affective similarity data across brain regions. The imputation was completed across all variables included in the models including variables of interest (condition, affective similarity, etc.), control variables (interaction quality, friendship quality, etc.) and nesting variables. The full dataset is available on databrary (Vallorani et al., 2023) and the full code is available on github (Vallorani, 2024).

Table S1. Clusters retained after whole-brain correction and subsetting against Neurosynth Mentalizing map

Cluster	Voxels	Center of Mass MNI Coordinates		
		x	y	z
dmPFC	757	0	55	21
rTPJ	375	51	-62	24
lTPJ	319	-50	-61	21
Precuneus	296	2	-57	36
vmPFC	154	-2	47	-18
Cerebellum 1	83	-23	-81	-37
Cerebellum 2	77	2	-53	-44

Note: dmPFC = Dorsal Medial Prefrontal Cortex,
rTPJ = Right Temporoparietal Junction,
lTPJ = Left Temporoparietal Junction,
vmPFC = Ventral Medial Prefrontal Cortex

Table S2. Multilevel model assessing contributions of condition, affective similarity and social anxiety symptoms to neural similarity with covariates

	Est	*SE*	*t*	*p*
Fixed Effects				
Intercept	-0.006	0.058	-0.10	.921
Discomfort Watching Self During BFFs Task	0.001	0.003	0.32	.753
Social Interaction Quality Visit 1	0.007	0.011	0.65	.541
Social Interaction Quality Visit 2	-0.010	0.010	-1.07	.287
Friendship Quality	0.011	0.010	1.14	.253
Trait Affective Similarity	-0.005	0.029	-0.18	.858
vmPFC	0.010	0.011	0.89	.373
rTPJ	0.002	0.011	0.15	.878
lTPJ	0.014	0.011	1.18	.237
State Affective Similarity	-0.008	0.005	-1.66	.097
Self-Perspective	0.007	0.010	0.70	.483
Friend-Perspective	0.000	0.010	0.02	.988
Social Anxiety	0.000	0.000	1.24	.217
State Affective Similarity x Self-Perspective	0.023	0.009	2.24	.015
State Affective Similarity x Friend-Perspective	-0.001	0.009	-0.06	.951
State Affective Similarity x Social Anxiety	0.000	0.000	0.85	.398
Self- Perspective x Social Anxiety	-0.001	0.001	-1.43	.153
Friend Perspective x Social Anxiety	0.000	0.000	-0.83	.407
State Affective Similarity x Self-Perspective x Social Anxiety	0.000	0.000	-0.17	.863
State Affective Similarity x Friend-Perspective x Social Anxiety	-0.001	0.000	-2.50	.013
Random Effects				
Dyad – Intercept	0.0008 (0.029)			
Dyad – Affective Similarity	0.0002 (0.016)			

Note: Pooled results across 25 imputed data sets with 9072 observations each.

Table S3. Multilevel model assessing contributions of condition and affective similarity to neural similarity

	Est	*SE*	*t*	*p*
Fixed Effects				
Intercept	0.014	0.042	0.33	.743
Trait Affective Similarity	-0.008	0.030	-0.28	.779
vmPFC	0.010	0.011	0.89	.372
rTPJ	0.002	0.011	0.15	.878
lTPJ	0.014	0.011	1.19	.235
State Affective Similarity	-0.008	0.005	-1.64	.102
Self-Perspective	0.006	0.010	0.56	.574
Friend-Perspective	-0.001	0.010	-0.06	.952
State Affective Similarity x Self-Perspective	0.024	0.009	2.51	.012
State Affective Similarity x Friend-Perspective	0.001	0.009	0.09	.927
Random Effects				
Dyad – Intercept	0.001 (0.033)			
Dyad – Affective Similarity	0.0002 (0.014)			

Note: Pooled results across 25 imputed data sets with 9072 observations each.

Table S4. Multilevel model assessing contributions of condition, affective similarity and social anxiety symptoms to neural similarity

	Est	*SE*	*t*	*p*
Fixed Effects				
Intercept	-0.019	0.042	-0.45	.662
Trait Affective Similarity	-0.030	0.030	-0.98	.343
vmPFC	0.009	0.012	0.72	.470
rTPJ	-0.002	0.012	-0.18	.857
lTPJ	0.013	0.012	1.07	.285
State Affective Similarity	-0.010	0.007	-1.44	.162
Self-View	0.019	0.011	1.73	.083
Friend-View	0.002	0.011	0.23	.821
Social Anxiety	0.001	0.000	1.41	.159
State Affective Similarity x Self-Perspective	0.027	0.010	2.80	.005
State Affective Similarity x Friend-Perspective	-0.002	0.009	-0.17	.861
State Affective Similarity x Social Anxiety	0.000	0.000	0.90	.373
Self-Perspective x Social Anxiety	-0.001	0.001	-0.92	.359
Friend-Perspective x Social Anxiety	-0.000	0.001	-0.87	.383
State Affective Similarity x Self-Perspective x Social Anxiety	-0.000	0.000	-0.27	.784
State Affective Similarity x Friend-Perspective x Social Anxiety	-0.002	0.000	-3.24	.001
Random Effects				
Dyad – Intercept	0.001 (0.034)			
Dyad – State Affective Similarity	0.0003 (0.018)			

Note: Model conducted over listwise deleted data (7616 observations).

Table S5. Multilevel model assessing interaction of condition and neural region on neural similarity

	Est	*SE*	*t*	*p*
Fixed Effects				
Intercept	0.025	0.014	1.80	.072
vmPFC	0.006	0.016	0.40	.689
rTPJ	-0.003	0.016	-0.18	.857
lTPJ	0.023	0.016	1.45	.148
Self-Perspective	0.005	0.020	0.24	.814
Friend-Perspective	0.002	0.020	0.12	.905
vmPFC x Self-Perspective	0.011	0.028	0.39	.699
rTPJ x Self-Perspective	0.023	0.028	0.82	.414
lTPJ x Self-Perspective	-0.028	0.028	-0.97	.332
vmPFC x Friend-Perspective	0.005	0.028	0.17	.866
rTPJ x Friend-Perspective	-0.003	0.028	-0.12	.904
lTPJ x Friend-Perspective	-0.012	0.028	-0.43	.664
Random Effects				
Dyad – Intercept	0.001 (0.032)			

Note: Pooled results across 25 imputed data sets with 9072 observations each.

Table S6. Multilevel model assessing contributions of condition, affective similarity and social anxiety symptoms to neural similarity in dmPFC

	Est	*Std. Est*	*SE*	*t*	*p*
Fixed Effects					
Intercept	0.053	-0.005	0.047	1.14	.281
Trait Affective Similarity	0.022	0.018	0.034	0.66	.523
State Affective Similarity	-0.008	-0.025	0.009	-0.85	.429
Self-Perspective	0.004	0.007	0.020	0.18	.856
Friend-Perspective	0.003	0.007	0.020	0.16	.876
Social Anxiety	0.001	0.045	0.001	1.41	.164
Affective Similarity x Self-Perspective	0.036	0.112	0.017	2.12	.043
Affective Similarity x Friend-Perspective	0.001	-0.002	0.017	0.07	.730
Affective Similarity x Social Anxiety	0.001	0.032	0.000	1.25	.246
Self-Perspective x Social Anxiety	-0.001	-0.041	0.001	-0.76	.445
Friend-Perspective x Social Anxiety	-0.000	-0.020	0.001	-0.39	.697
Affective Similarity x Self-Perspective x Social Anxiety	-0.001	-0.037	0.001	-0.67	.529
Affective Similarity x Friend-Perspective x Social Anxiety	-0.001	-0.069	0.001	-1.33	.213
Random Effects					
Dyad – Intercept	0.001 (0.024)				
Dyad – Affective Similarity	0.000 (0.016)				

Note: Pooled results across 25 imputed data sets with 2268 observations each.

Table S7. Multilevel model assessing contributions of condition, affective similarity and social anxiety symptoms to neural similarity in rTPJ

	Est	*Std. Est*	*SE*	*t*	*p*
Fixed Effects					
Intercept	0.010	-0.018	0.055	0.18	.857
Trait Affective Similarity	-0.007	-0.012	0.040	-0.19	.850
State Affective Similarity	-0.015	-0.042	0.010	-1.60	.126
Self-Perspective	0.028	0.069	0.021	1.35	.176
Friend-Perspective	0.001	-0.004	0.020	0.06	.947
Social Anxiety	0.001	0.048	0.001	1.42	.157
Affective Similarity x Self-Perspective	0.033	0.097	0.018	1.88	.081
Affective Similarity x Friend-Perspective	0.007	0.014	0.017	0.39	.686
Affective Similarity x Social Anxiety	0.000	0.019	0.000	0.69	.490
Self-Perspective x Social Anxiety	-0.001	-0.036	0.001	-0.62	.534
Friend-Perspective x Social Anxiety	-0.002	-0.106	0.001	-2.14	.033
Affective Similarity x Self-Perspective x Social Anxiety	-0.000	-0.007	0.001	-0.15	.700
Affective Similarity x Friend-Perspective x Social Anxiety	-0.001	-0.082	0.001	-1.56	.149
Random Effects					
Dyad – Intercept	0.001 (0.035)				
Dyad – Affective Similarity	0.000 (0.018)				

Note: Pooled results across 25 imputed data sets with 2268 observations each.

Table S8. Bayesian multilevel model assessing contributions of condition, affective similarity and social anxiety symptoms to neural similarity

	Est	Est.Error	Lower 95% CI	Upper95% CI
Intercept	0.01	0.05	-0.08	0.10
Trait Affective Similarity	-0.01	0.03	-0.08	0.05
vmPFC	0.01	0.01	-0.01	0.03
rTPJ	0.00	0.01	-0.02	0.02
lTPJ	0.01	0.01	-0.01	0.04
State Affective Similarity	-0.01	0.00	-0.02	0.00
Self-Perspective	0.01	0.01	-0.01	0.03
Friend-Perspective	0.00	0.01	-0.02	0.02
Social Anxiety	0.00	0.00	-0.00	0.00
Affective Similarity x Self-Perspective	0.02	0.01	0.00	0.04
Affective Similarity x Friend-Perspective	-0.00	0.01	-0.02	0.02
Affective Similarity x Social Anxiety	0.00	0.00	-0.00	0.00
Self-Perspective x Social Anxiety	-0.00	0.00	-0.00	0.00
Friend-Perspective x Social Anxiety	-0.00	0.00	-0.00	0.00
Affective Similarity x Self-Perspective x Social Anxiety	-0.00	0.00	-0.00	0.00
Affective Similarity x Friend-Perspective x Social Anxiety	-0.00	0.00	-0.00	-0.00

Note: Pooled results across 25 imputed data sets with 9072 observations each.

Table S9. Bayesian multilevel model assessing contributions of condition and affective similarity to neural similarity

	Est	Est.Error	Lower 95% CI	Upper 95% CI
Intercept	0.01	0.05	-0.08	0.11
Trait Affective Similarity	-0.01	0.03	-0.07	0.06
vmPFC	0.01	0.01	-0.01	0.03
rTPJ	0.00	0.01	-0.02	0.02
lTPJ	0.01	0.01	-0.01	0.04
State Affective Similarity	-0.01	0.00	-0.02	0.00
Self-Perspective	0.01	0.01	-0.01	0.03
Friend-Perspective	-0.00	0.01	-0.02	0.02
State Affective Similarity x Self-Perspective	0.02	0.01	0.01	0.04
State Affective Similarity x Friend-Perspective	0.00	0.01	-0.02	0.02

Note: Pooled results across 25 imputed data sets with 9072 observations each.

Table S10. Bayesian multilevel model assessing interaction of condition and neural region on neural similarity

	Est.	Est. Error	Lower 95% CI	Upper-95% CI
Intercept	0.02	0.01	-0.00	0.05
vmPFC	0.01	0.02	-0.02	0.04
rTPJ	-0.00	0.02	-0.03	0.03
lTPJ	0.02	0.02	-0.01	0.05
Self-Perspective	0.00	0.02	-0.03	0.04
Friend-Perspective	0.00	0.02	-0.04	0.04
vmPFC x Self-Perspective	0.01	0.03	-0.04	0.07
rTPJ x Self-Perspective	0.02	0.03	-0.03	0.08
lTPJ x Self-Perspective	-0.03	0.03	-0.08	0.03
vmPFC x Friend-Perspective	0.00	0.03	-0.05	0.06
rTPJ x Friend-Perspective	-0.00	0.03	-0.06	0.05
lTPJ x Friend-Perspective	-0.01	0.03	-0.07	0.04

Note: Pooled results across 25 imputed data sets with 9072 observations each.

Table S11. Bayesian multilevel model assessing contributions of condition, affective similarity and social anxiety symptoms to neural similarity in dmPFC

	Est.	Est. Error	Lower 95% CI	Upper 95% CI
Intercept	0.05	0.05	-0.05	0.16
Trait Affective Similarity	0.02	0.04	-0.05	0.10
State Affective Similarity	-0.01	0.01	-0.03	0.01
Self-Perspective	0.00	0.02	-0.04	0.04
Friend-Perspective	0.00	0.02	-0.04	0.04
Social Anxiety	0.00	0.00	-0.00	0.00
Affective Similarity x Self-Perspective	0.04	0.02	0.00	0.07
Affective Similarity x Friend-Perspective	0.00	0.02	-0.03	0.04
Affective Similarity x Social Anxiety	0.00	0.00	-0.00	0.00
Self-Perspective x Social Anxiety	-0.00	0.00	-0.00	0.00
Friend-Perspective x Social Anxiety	-0.00	0.00	-0.00	0.00
Affective Similarity x Self-Perspective x Social Anxiety	-0.00	0.00	-0.00	0.00
Affective Similarity x Friend-Perspective x Social Anxiety	-0.00	0.00	-0.00	0.00

Note: Pooled results across 25 imputed data sets with 2268 observations each.

Table S12. Multilevel model assessing contributions of condition, affective similarity and social anxiety symptoms to neural similarity in rTPJ

	Est.	Est. Error	Lower 95% CI	Upper 95% CI
Intercept	0.01	0.06	-0.11	0.13
Trait Affective Similarity	-0.01	0.04	-0.09	0.08
State Affective Similarity	-0.02	0.01	-0.03	0.00
Self-Perspective	0.03	0.02	-0.01	0.07
Friend-Perspective	0.00	0.02	-0.04	0.04
Social Anxiety	0.00	0.00	-0.00	0.00
Affective Similarity x Self-Perspective	0.03	0.02	-0.00	0.07
Affective Similarity x Friend-Perspective	0.01	0.02	-0.03	0.04
Affective Similarity x Social Anxiety	0.00	0.00	-0.00	0.00
Self-Perspective x Social Anxiety	-0.00	0.00	-0.00	0.00
Friend-Perspective x Social Anxiety	-0.00	0.00	-0.00	-0.00
Affective Similarity x Self-Perspective x Social Anxiety	-0.00	0.00	-0.00	0.00
Affective Similarity x Friend-Perspective x Social Anxiety	-0.00	0.00	-0.00	0.00

Note: Pooled results across 25 imputed data sets with 2268 observations each.

Table S13: Simple slopes analysis of two-way interaction for all regions

Affective Similarity	Condition	*Estimate*	*SE*	*t*	*p*
-1.19 (-1 *SD*)	Self-Perspective	-0.014	0.016	-0.888	0.374
-1.19 (-1 *SD*)	Friend-Perspective	0.000	0.015	-0.016	0.988
0 (*M*)	Self-Perspective	0.018	0.011	1.648	0.099
0 (*M*)	Friend-Perspective	0.002	0.011	0.146	0.884
**1.19 (+1 *SD*)**	**Self-Perspective**	**0.051**	**0.015**	**3.289**	**0.001**
1.19 (+1 *SD*)	Friend-Perspective	0.003	0.015	0.217	0.828

Note: SD = standard deviation, *M* = mean, *SE* = Standard Error

Table S14. Simple slopes analysis of three-way interaction for all regions

Affective Similarity	Condition	Social Anxiety Symptoms	*Estimate*	*SE*	*t*	*p*
-1.19 (-1 *SD*)	Self-Perspective	-18.41 (-1 *SD*)	-0.005	0.024	-0.229	0.819
-1.19 (-1 *SD*)	Friend-Perspective	-18.41 (-1 *SD*)	-0.020	0.020	0.989	0.323
0 (*M*)	Self-Perspective	-18.41 (-1 *SD*)	0.029	0.016	1.775	0.076
0 (*M*)	Friend-Perspective	-18.41 (-1 *SD*)	0.011	0.015	0.752	0.452
**1.19 (+1 *SD*)**	**Self-Perspective**	**-18.41 (-1 *SD*)**	**0.064**	**0.023**	**2.814**	**0.005**
**1.19 (+1 *SD*)**	**Friend-Perspective**	**-18.41 (-1 *SD*)**	**0.042**	**0.021**	**2.027**	**0.043**
-1.19 (-1 *SD*)	Self-Perspective	1.24 (*M*)	-0.013	0.016	-0.793	0.428
-1.19 (-1 *SD*)	Friend-Perspective	1.24 (*M*)	0.006	0.015	0.394	0.693
0 (*M*)	Self-Perspective	1.24 (*M*)	0.019	0.011	1.687	0.092
0 (*M*)	Friend-Perspective	1.24 (*M*)	0.002	0.011	0.173	0.863
**1.19 (+1 *SD*)**	**Self-Perspective**	**1.24 (*M*)**	**0.050**	**0.015**	**3.250**	**0.001**
1.19 (+1 *SD*)	Friend-Perspective	1.24 (*M*)	-0.002	0.016	-0.145	0.884
-1.19 (-1 *SD*)	Self-Perspective	20.89 (+1 *SD*)	-0.020	0.022	-0.916	0.360
-1.19 (-1 *SD*)	Friend-Perspective	20.89 (+1 *SD*)	0.032	0.023	1.408	0.159
0 (*M*)	Self-Perspective	20.89 (+1 *SD*)	0.008	0.015	0.538	0.591
0 (*M*)	Friend-Perspective	20.89 (+1 *SD*)	-0.007	0.015	-0.480	0.632
1.19 (+1 *SD*)	Self-Perspective	20.89 (+1 *SD*)	0.037	0.022	1.693	0.090
**1.19 (+1 *SD*)**	**Friend-Perspective**	**20.89 (+1 *SD*)**	**-0.047**	**0.023**	**-2.058**	**0.040**

Note: SD = standard deviation, *M* = mean, *SE* = Standard Error

Table S15. Simple slopes analysis of two-way interaction for dmPFC

Social Anxiety Symptoms	Condition	*Estimate*	*SE*	*t*	*p*
-18.59 (-1 *SD*)	Self-Perspective	0.041	0.030	1.359	0.174
-18.59 (-1 *SD*)	Friend-Perspective	0.041	0.028	1.460	0.144
1.33 (*M*)	Self-Perspective	0.028	0.021	1.349	0.178
1.33 (*M*)	Friend-Perspective	-0.002	0.020	-0.089	0.929
21.25 (+1 *SD*)	Self-Perspective	0.015	0.029	0.506	0.613
21.25 (+1 *SD*)	Friend-Perspective	-0.044	0.029	-1.541	0.123

Note: SD = standard deviation, *M* = mean, *SE* = Standard Error

Table S16. Simple slopes analysis of two-way interaction for rTPJ

Affective Similarity	Condition	*Estimate*	*SE*	*t*	*p*
-1.19 (-1 *SD*)	Self-Perspective	-0.024	0.032	-0.730	0.465
-1.19 (-1 *SD*)	Friend-Perspective	0.013	0.030	0.446	0.656
0 (*M*)	Self-Perspective	0.023	0.022	1.057	0.291
0 (*M*)	Friend-Perspective	0.016	0.022	0.722	0.470
**1.19 (+1 *SD*)**	**Self-Perspective**	**0.070**	**0.031**	**2.274**	**0.023**
1.19 (+1 *SD*)	Friend-Perspective	0.018	0.031	0.569	0.569

Note: SD = standard deviation, *M* = mean, *SE* = Standard Error

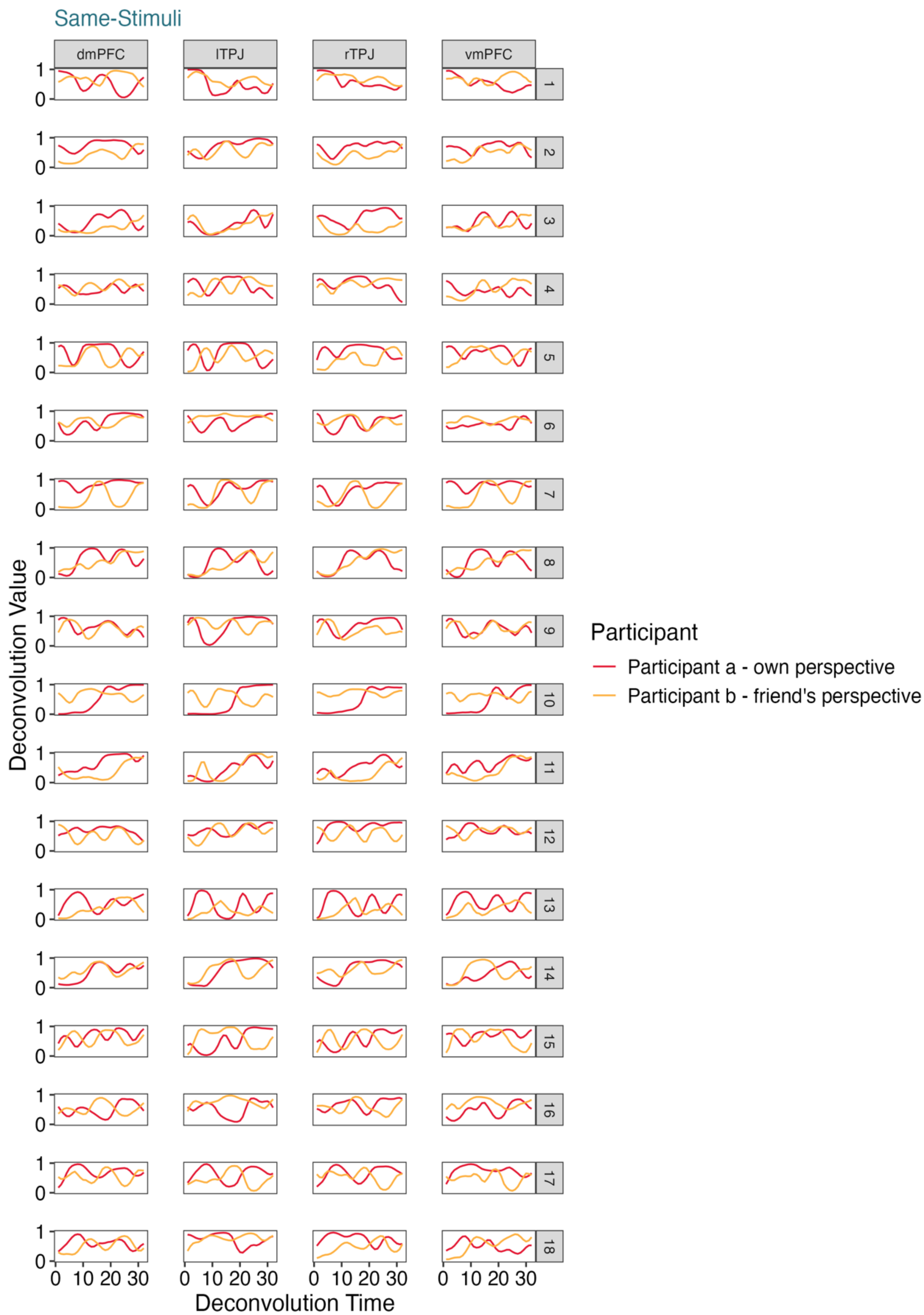

Figure S1. Neural signal for Same-Stimuli condition where Participant a viewed from their own perspective and Participant b viewed from their friend's perspective

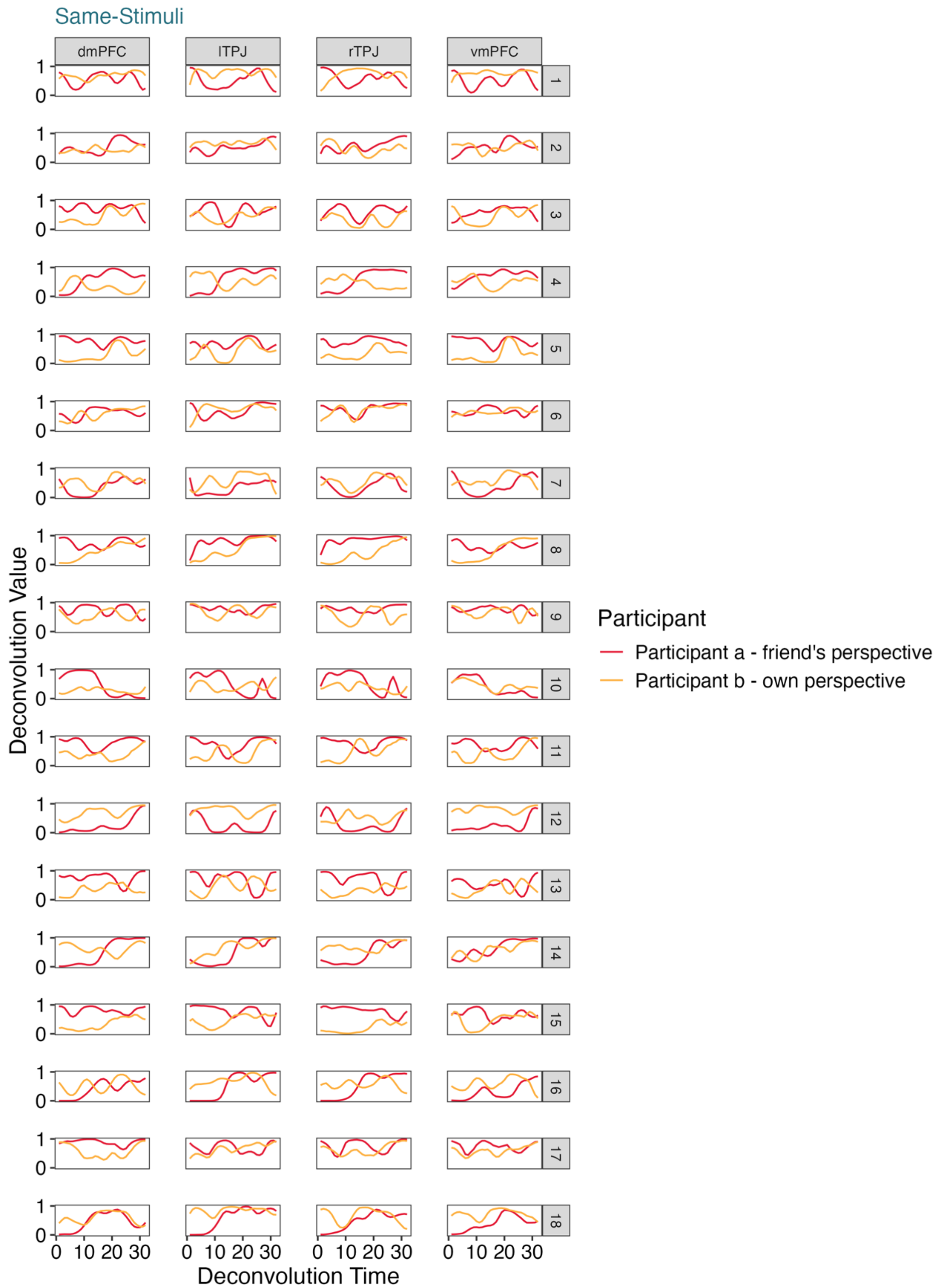

Figure S2. Neural signal for Same-Stimuli condition where Participant a viewed from their friend's perspective and Participant b viewed from their own perspective

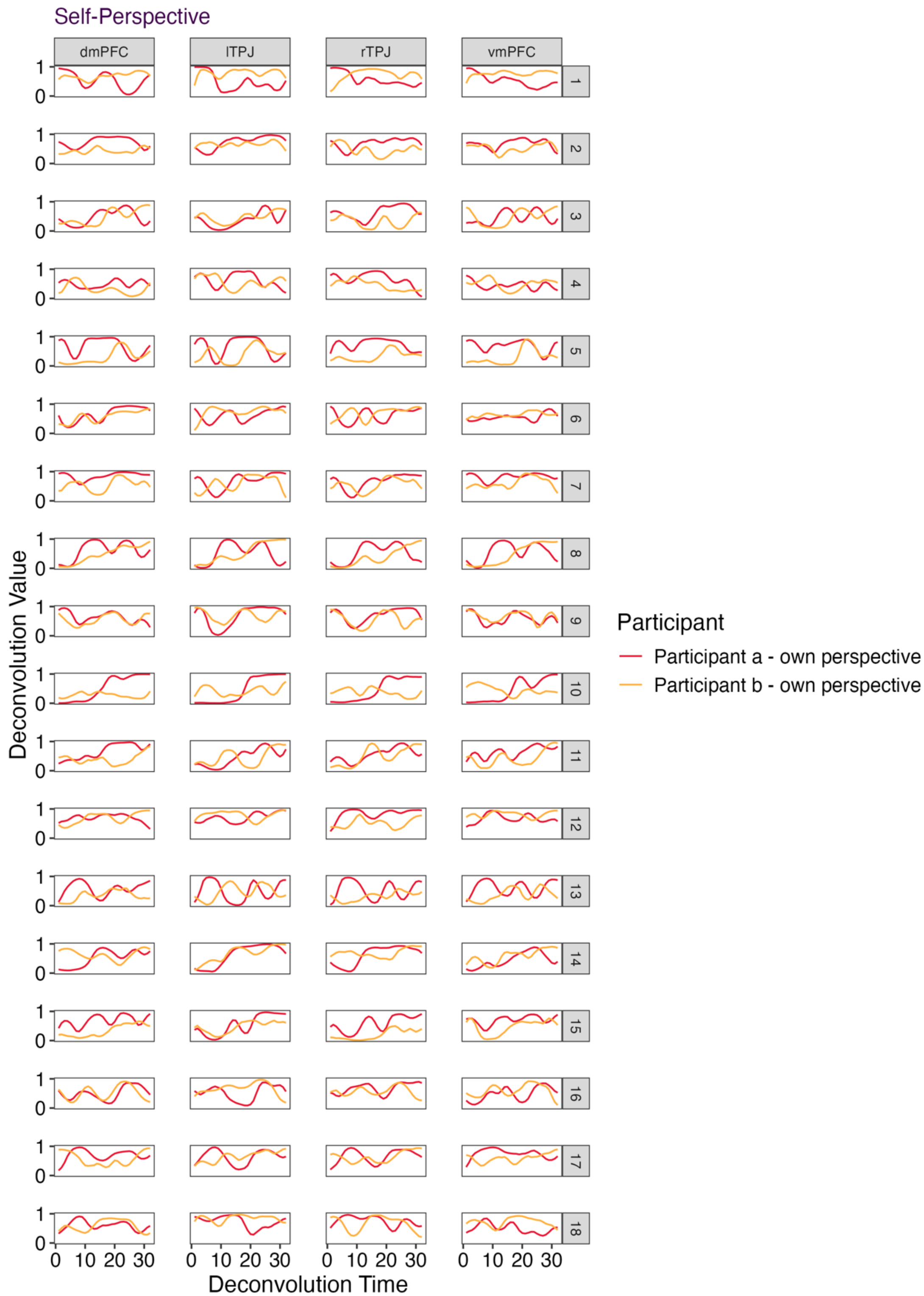

Figure S3. Neural signal for Self-Perspective condition where both participants viewed from their own perspective.

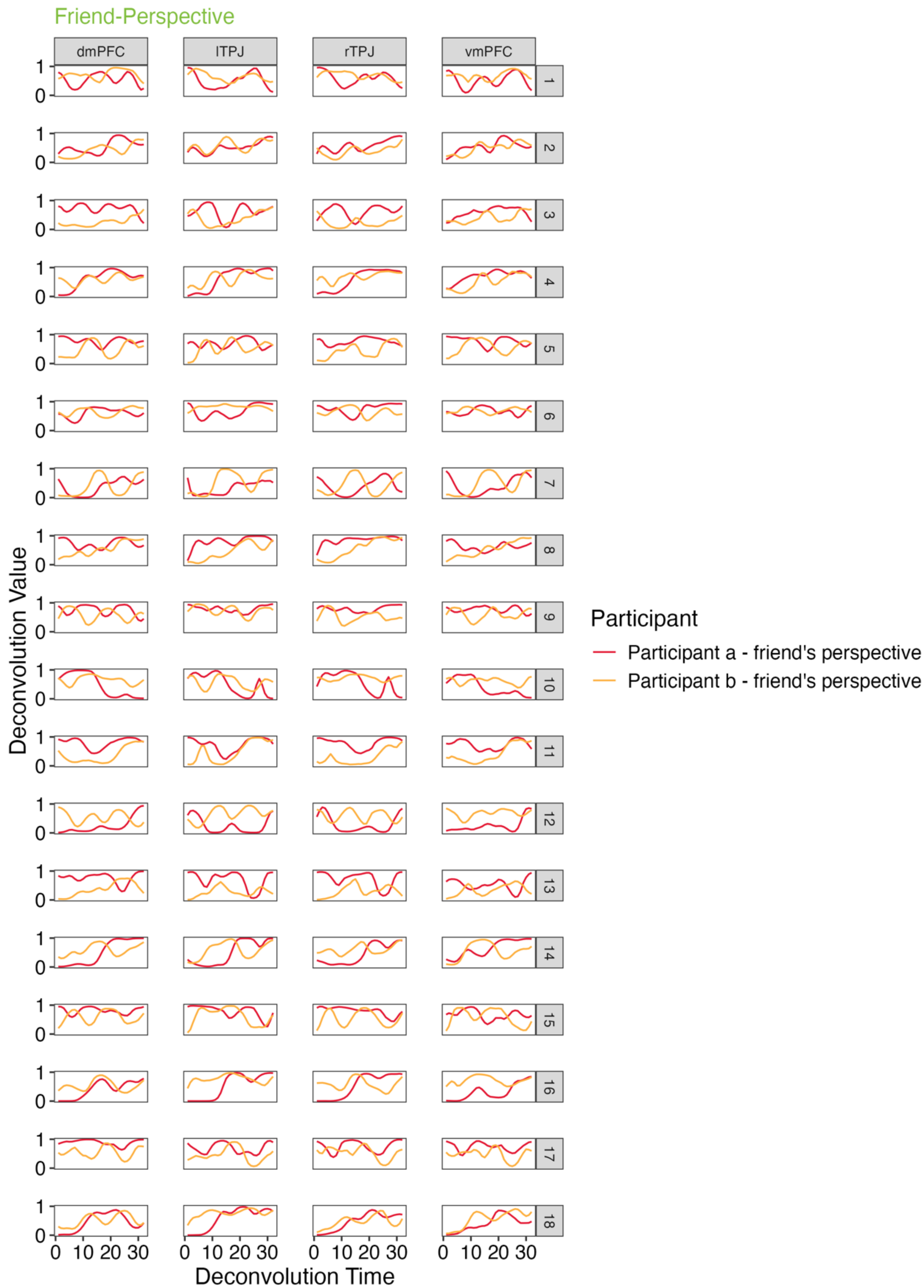

Figure S4. Neural signal for Self-Perspective condition where both participants viewed from their own perspective.

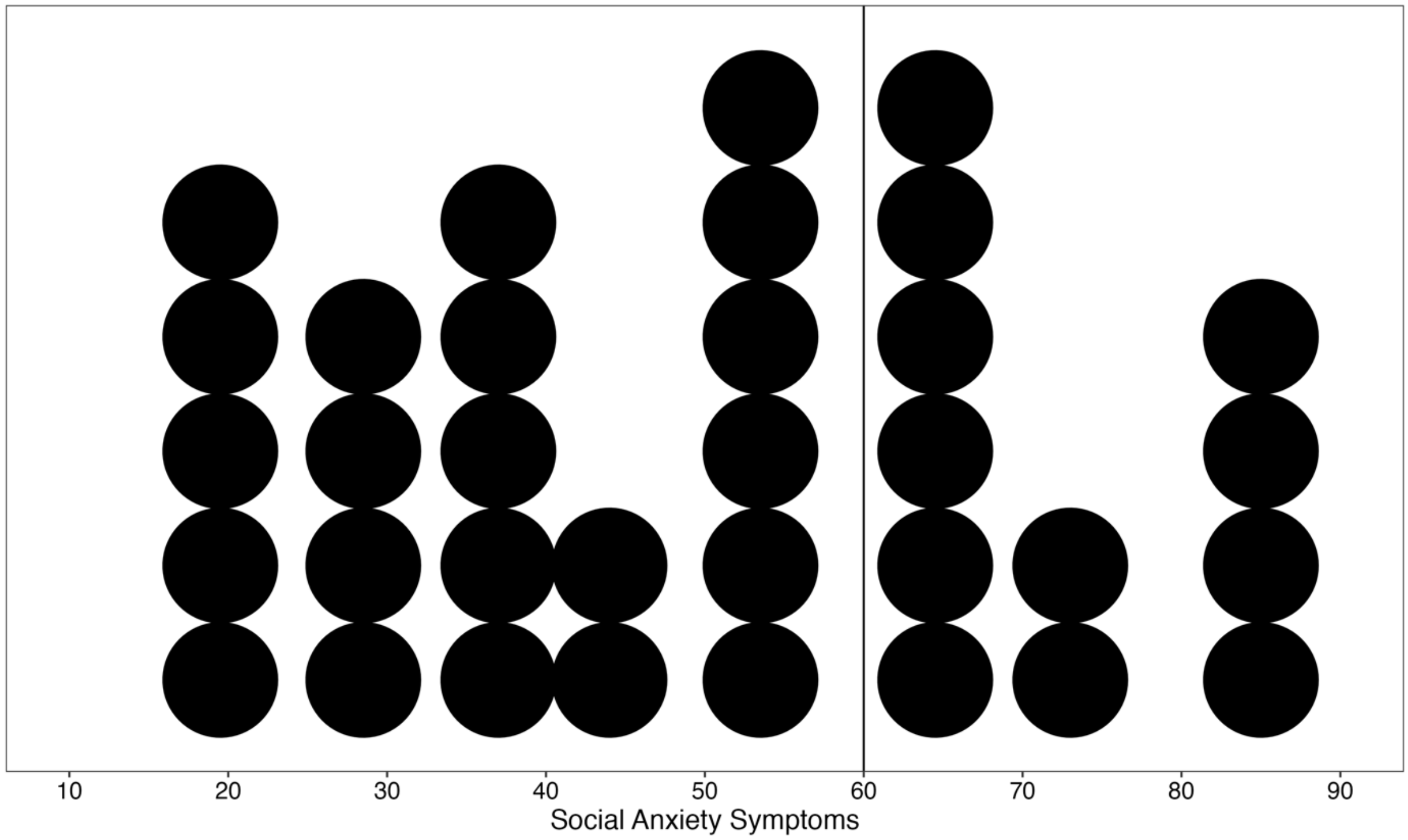

Figure S5. Distribution of social anxiety symptoms. Included participants exhibited a range of social anxiety scores and 12 out of 34 participants exhibited scores higher than 60, a common cutoff for social anxiety (Mennin et al., 2002).

References

Abraham, A., Pedregosa, F., Eickenberg, M., Gervais, P., Mueller, A., Kossaifi, J., Gramfort, A., Thirion, B., & Varoquaux, G. (2014). Machine learning for neuroimaging with scikit-learn. *Frontiers in Neuroinformatics*, *8*. https://doi.org/10.3389/fninf.2014.00014

Andersson, J. L. R., Skare, S., & Ashburner, J. (2003). How to correct susceptibility distortions in spin-echo echo-planar images: Application to diffusion tensor imaging. *NeuroImage*, *20*(2), 870–888. https://doi.org/10.1016/S1053-8119(03)00336-7

Avants, B., Epstein, C., Grossman, M., & Gee, J. (2008). Symmetric diffeomorphic image registration with cross-correlation: Evaluating automated labeling of elderly and neurodegenerative brain. *Medical Image Analysis*, *12*(1), 26–41. https://doi.org/10.1016/j.media.2007.06.004

Behzadi, Y., Restom, K., Liau, J., & Liu, T. T. (2007). A component based noise correction method (CompCor) for BOLD and perfusion based fMRI. *NeuroImage*, *37*(1), 90–101. https://doi.org/10.1016/j.neuroimage.2007.04.042

Ciric, R., Thompson, W. H., Lorenz, R., Goncalves, M., MacNicol, E. E., Markiewicz, C. J., Halchenko, Y. O., Ghosh, S. S., Gorgolewski, K. J., Poldrack, R. A., & Esteban, O. (2022). TemplateFlow: FAIR-sharing of multi-scale, multi-species brain models. *Nature Methods*, *19*(12), 1568–1571. https://doi.org/10.1038/s41592-022-01681-2

Dale, A. M., Fischl, B., & Sereno, M. I. (1999). Cortical surface-based analysis. *NeuroImage*, *9*(2), 179–194. https://doi.org/10.1006/nimg.1998.0395

Fonov, V., Evans, A., McKinstry, R., Almli, C., & Collins, D. (2009). Unbiased nonlinear average age-appropriate brain templates from birth to adulthood. *NeuroImage*, *47*, S102. https://doi.org/10.1016/S1053-8119(09)70884-5

Greve, D. N., & Fischl, B. (2009). Accurate and robust brain image alignment using boundary-based registration. *NeuroImage*, *48*(1), 63–72. https://doi.org/10.1016/j.neuroimage.2009.06.060

Jenkinson, M., Bannister, P., Brady, M., & Smith, S. (2002). Improved optimization for the robust and accurate linear registration and motion correction of brain images. *NeuroImage*, *17*(2), 825–841. https://doi.org/10.1006/nimg.2002.1132

Klein, A., Ghosh, S. S., Bao, F. S., Giard, J., Häme, Y., Stavsky, E., Lee, N., Rossa, B., Reuter, M., Chaibub Neto, E., & Keshavan, A. (2017). Mindboggling morphometry of human brains. *PLOS Computational Biology*, *13*(2), e1005350. https://doi.org/10.1371/journal.pcbi.1005350

Lanczos, C. (1964). Evaluation of Noisy Data. *Journal of the Society for Industrial and Applied Mathematics Series B Numerical Analysis*, *1*(1), 76–85. https://doi.org/10.1137/0701007

Mennin, D. S., Fresco, D. M., Heimberg, R. G., Schneier, F. R., Davies, S. O., & Liebowitz, M. R. (2002). Screening for social anxiety disorder in the clinical setting: Using the Liebowitz Social Anxiety Scale. *Journal of Anxiety Disorders*, *16*(6), 661–673. https://doi.org/10.1016/S0887-6185(02)00134-2

Patriat, R., Reynolds, R. C., & Birn, R. M. (2017). An improved model of motion-related signal changes in fMRI. *NeuroImage*, *144*, 74–82. https://doi.org/10.1016/j.neuroimage.2016.08.051

Power, J. D., Mitra, A., Laumann, T. O., Snyder, A. Z., Schlaggar, B. L., & Petersen, S. E. (2014). Methods to detect, characterize, and remove motion artifact in resting state fMRI. *NeuroImage*, *84*, 320–341. https://doi.org/10.1016/j.neuroimage.2013.08.048

Pruim, R. H. R., Mennes, M., van Rooij, D., Llera, A., Buitelaar, J. K., & Beckmann, C. F. (2015). ICA-AROMA: A robust ICA-based strategy for removing motion artifacts from fMRI data. *NeuroImage*, *112*, 267–277. https://doi.org/10.1016/j.neuroimage.2015.02.064

Satterthwaite, T. D., Elliott, M. A., Gerraty, R. T., Ruparel, K., Loughead, J., Calkins, M. E., Eickhoff, S. B., Hakonarson, H., Gur, R. C., Gur, R. E., & Wolf, D. H. (2013). An improved framework for confound regression and filtering for control of motion artifact in the preprocessing of resting-state functional connectivity data. *NeuroImage*, *64*, 240–256. https://doi.org/10.1016/j.neuroimage.2012.08.052

Tustison, N. J., Avants, B. B., Cook, P. A., Yuanjie Zheng, Egan, A., Yushkevich, P. A., & Gee, J. C. (2010). N4ITK: Improved N3 Bias Correction. *IEEE Transactions on Medical Imaging*, *29*(6), 1310–1320. https://doi.org/10.1109/TMI.2010.2046908

Vallorani, A. (2024). *BFFs*. https://github.com/auv27/BFFs

Vallorani, A., Lytel, M. N., & Pérez-Edgar, K. (2023). *Behavioral Foundations of Friendship Study (BFFs)*. https://nyu.databrary.org/volume/1609

Zhang, Y., Brady, M., & Smith, S. (2001). Segmentation of brain MR images through a hidden Markov random field model and the expectation-maximization algorithm. *IEEE Transactions on Medical Imaging*, *20*(1), 45–57. https://doi.org/10.1109/42.906424
